# Diversity and evolution of an abundant ICE*clc* family of integrative and conjugative elements in *Pseudomonas aeruginosa*

**DOI:** 10.1128/msphere.00517-23

**Published:** 2023-10-30

**Authors:** Valentina Benigno, Nicolas Carraro, Garance Sarton-Lohéac, Sara Romano-Bertrand, Dominique S. Blanc, Jan Roelof van der Meer

**Affiliations:** 1 Department of Fundamental Microbiology, University of Lausanne, Lausanne, Switzerland; 2 Hydrosciences Montpellier, IRD, CNRS, University of Montpellier, Hospital Hygiene and Infection Control Team, University Hospital of Montpellier, Montpellier, France; 3 Prevention and Infection Control Unit, Infectious Diseases Service, Lausanne University Hospital and University of Lausanne, Lausanne, Switzerland; Escola Paulista de Medicina/Universidade Federal de São Paulo, São Paulo, Brazil

**Keywords:** integrative and conjugative element, *clc* element, bacterial evolution, *Pseudomonas aeruginosa*, horizontal gene transfer, adaptation

## Abstract

Integrative and conjugative elements (ICEs) are widespread autonomous mobile DNA elements, containing the genes necessary for their excision, conjugative transfer, and insertion into a new host cell. ICEs can carry additional genes that are non-essential for their transfer but can confer adaptive phenotypes to the host. Our aim here was to better characterize the presence, distribution, and variation of ICEs related to the well-described ICE*clc* among *Pseudomonas aeruginosa* clinical isolates within a geographically restrained environment to understand the factors contributing to their evolution. We examined a total of 181 *P*. *aeruginosa* genome sequences obtained from patient or hospital environment isolates, most of which were obtained from a single hospital during 20 years of sampling. More than 90% of the isolates carried one or more ICE*clc*-like elements, with different degrees of conservation to the known ICE*clc* lifestyle and transfer genes. ICE clones closely matched their host clonal phylogeny, but not exclusively, indicating that both clonal evolution and ICE horizontal transfer are occurring in the hospital environment. ICEs from this singular hospital environment were mainly associated to three clone types found worldwide, suggesting an enrichment of local clones. Variable gene regions among the clinical *P. aeruginosa* ICE*clc*-type elements were notably enriched for heavy metal resistance genes, toxin-anti-toxin systems, potential efflux systems and multidrug resistance proteins, a metalloprotease and for a variety of regulatory systems, but not for specific recognizable antibiotic-resistance cassettes. Clonal persistence suggests adaptive benefits of these functional categories, and micro-patterns of gene gain and loss indicate ongoing ICE evolution within the *P. aeruginosa* hosts.

**IMPORTANCE:**

Microbial populations swiftly adapt to changing environments through horizontal gene transfer. While the mechanisms of gene transfer are well known, the impact of environmental conditions on the selection of transferred gene functions remains less clear. We investigated ICEs, specifically the ICE*clc*-type, in *Pseudomonas aeruginosa* clinical isolates. Our findings revealed co-evolution between ICEs and their hosts, with ICE transfers occurring within strains. Gene functions carried by ICEs are positively selected, including potential virulence factors and heavy metal resistance. Comparison to publicly available *P. aeruginosa* genomes unveiled widespread antibiotic-resistance determinants within ICE*clc* clades. Thus, the ubiquitous ICE*clc* family significantly contributes to *P. aeruginosa*’s adaptation and fitness in diverse environments.

## INTRODUCTION

*Pseudomonas aeruginosa* is a ubiquitous bacterium found in a diversity of environments, from soil to water, and can act as an opportunistic pathogen of a wide range of hosts including humans (1). *P. aeruginosa* isolates are well known as a major cause of infections in the cystic fibrosis lung (2) and are further responsible for the occurrence of nosocomial diseases, especially in intensive care units (3). The remarkable capability of *P. aeruginosa* to colonize a wide range of environments and hosts is reflected in its large genome with a high proportion of regulatory genes (4). Additionally, genomes of *P. aeruginosa* are mosaics of conserved core parts interspersed with highly variable accessory elements (5–8). The core genome, defined as the set of genes that are present in nearly all strains of *P. aeruginosa*, has an average inter-strain diversity of 0.5%–0.7% and makes up some 90% of the total genome of each strain (6, 9–11). The accessory genome consists of extrachromosomal elements like plasmids, as well as segments of DNA variable in size and content that are inserted into the chromosome at various loci (12–14). The most important process contributing to the evolution of the *P. aeruginosa* accessory genome is thought to be horizontal gene transfer (14).

The chromosomal loci where accessory genomic segments are frequently integrated have been called “regions of genome plasticity” (15). Some of these variable regions occur nearby genes coding for tRNAs and bear signatures of integrative and conjugative elements (ICEs) (14). ICEs are widespread mobile DNA elements in bacteria (16–20) that remain by integration in the host chromosome but can excise to form an autonomous molecule that can be conjugated to and inserted into the chromosome of new recipients (21). The notion of wide ICE distribution originated from a study that quantified the abundance and diversity of conjugative systems among prokaryotes, at that time covering 1,124 complete genomes (16). Notably, that study showed that all bacterial clades for which a significant number of sequenced genomes were available encode conjugative systems in their chromosomes. Classification of conserved conjugative marker genes suggested important abundance of ICEs, as well as conjugative plasmids (16). ICEs not only encode all the genes necessary for their integration, excision, and the conjugative machinery but also contain “cargo” genes that can confer adaptive phenotypes to the host (22). Known examples include antibiotic or heavy metal resistance, or the ability to metabolize specific or xenobiotic carbon sources (23–25).

Previously characterized ICEs in *P. aeruginosa* include elements related not only to ICE*clc* (of the taxonomic relative *Pseudomonas knackmussii* B13) (26) but also to PAGI-2 (in strain C), PAGI-3 (in strain SG17M), LESGI-3 (in strain LESB58) (5, 14), and PAPI-1 (in strain PA14) (8). The described *P. aeruginosa* ICEs share a set of highly conserved genes, while the primary difference between them is their cargo genes (12, 27). The ICE*clc* family is a loosely defined group of elements detected among Beta- and Gammaproteobacterial genomes (17, 28) with ICE*clc* as the main studied representative. ICE*clc* is 103 kb in size, occurs in two identical copies in the *P. knackmussii* B13 genome (28), and is characterized by the presence of the *clc* genes for chlorocatechol degradation (25). The element can transfer readily from strain B13 to *P. aeruginosa* (29) and to other recipients belonging to Beta- and Gammaproteobacteria lineages (30, 31). In addition to the *clc* genes themselves, ICE*clc* further carries genes for 2-aminophenol degradation (25), for a multidrug efflux pump (32), as well as for additional proteins of unknown function. A 50-kb region on ICE*clc*, with high sequence similarity to other (suspected) ICEs (25, 28, 33), contains the genes for ICE activation (33, 34), excision and integration (35), DNA processing (36) and conjugative transfer (37). A wide variety of ICE*clc* family elements have been identified in different bacterial hosts, and more recent surveys point to several instances of relatives carrying antibiotic-resistance determinants in clinical isolates (26, 38–43). In particular, the studies by Botelho et al. (38) and Hong et al. (42) showed ICEs related to ICE*clc* encoding carbapenem resistance in *P. aeruginosa*. These studies thus indicated that ICEs of the ICE*clc* family appear in various hosts and geographical locations worldwide and with different (adaptive) gene content. However, since they largely occur in very different isolates, it is difficult to understand ongoing ICE evolution and adaptation.

The main objective of our work was to study the micro-evolution of ICEs related to the ICE*clc* family over time within a coherent environment and to find potential evidence for enrichment of adaptive gene functions and selection. For this, we focused on clinical strains of *P. aeruginosa* isolated from patients and the hospital environment itself, across a period of 20 years. We included mainly isolates from a single hospital (Lausanne University Hospital) but also from nearby hospitals, with the idea that regional transmission might be detectable (Geneva, Montpellier, and Besançon). Genome sequences of 181 isolates were assembled, among which we searched for ICEs of the ICE*clc* family. On a smaller subset of non-redundant ICEs, we delineated the potential ICE boundaries wherever possible and analyzed conserved and variable (adaptive) gene contents within defined ICE regions. ICE and host phylogenies were compared from all or from single-copy orthologous genes, in order to delineate potential vertical and horizontal ICE transmission events. Local ICEs were then further phylogenetically compared to a set of recently identified ICEs among *P. aeruginosa* isolates in a worldwide survey (44). Selection for ICE adaptive functions was analyzed by comparison to random models, suggesting significant enrichment of a variety of gene functions in the local ICE pool, but (so far) not of specific antibiotic-resistance genes, although these are circulating in the worldwide pool.

## RESULTS

### *Pseudomonas aeruginosa* clinical isolates contain an abundance of ICE*clc*-like elements

To describe the micro-evolution of ICEs and, in particular, of ICE*clc*-type elements, we analyzed 181 *P*. *aeruginosa* genomes, obtained from strains isolated between 2003 and 2022 in a geographically constrained area. Most isolates originated from the Lausanne University Hospital (CHUV), further complemented with isolates from the Geneva University Hospital (HUG), University Hospital of Montpellier (CHU Montpellier), and Besançon Regional University Hospital Center (CHU Besançon), to survey potential regional transmission. The strains were isolated either from the hospital environment (e.g., contaminated hand soaps and door handles) or from infected patients (Table S1). Genomes were constructed by *de novo* assembly followed by reference-guided contigs scaffolding and patching (deploying the reference *P. aeruginosa* strain H26027 genome; National Center for Biotechnology Information (NCBI) accession number CP033684). The scaffolds have on average 69 gaps (Table S1). Strain genotyping by multilocus sequence typing (MLST, Table S1) showed that some of the analyzed strains belong to the epidemic high-risk clones (namely, ST395 and ST111) (45), which are characterized by frequent integron- or transposon-mediated acquisition of antibiotic-resistance elements (46). As expected, some MLST types are overrepresented in our data set (Table S1), and the strain collection represents a restricted set of the global distribution of sequence types (44).

**Fig 1:**
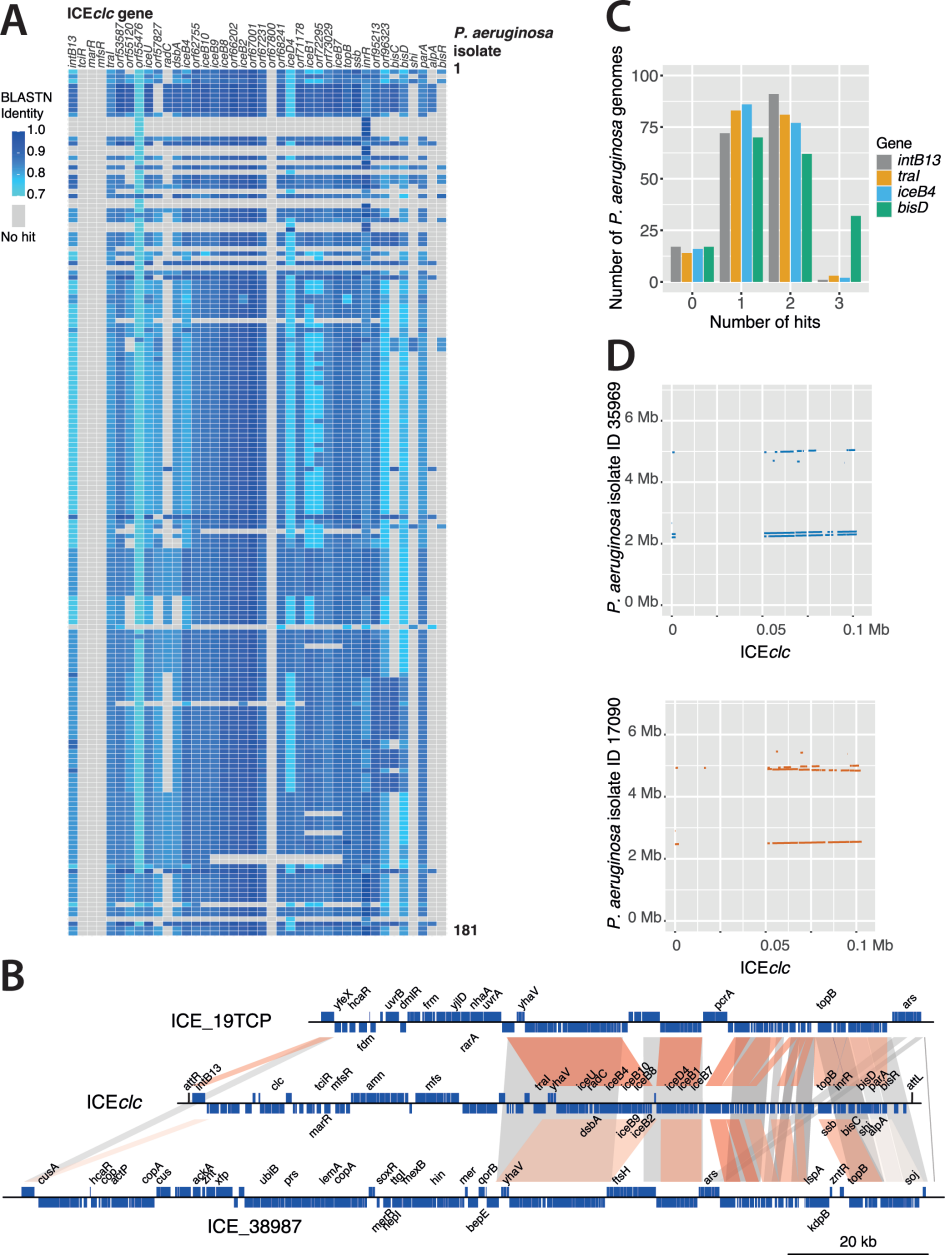
ICE*clc*-like elements are abundant among *P. aeruginosa* clinical isolates. (**A**) BLASTN similarities (as fraction of 1 = 100%, blue hue color legend) for 40 ICE*clc* genes with 181 *P*. *aeruginosa* draft genomes (single top hit per ICE*clc* per genome, similarity over full gene lengths). ICE*clc* queries included *intB13* integrase, *traI* relaxase, DNA topoisomerase *topB*, 13 ORFs of the regulation module, and 24 ORFs whose products form the type IV secretion system (ORF or gene names on top, horizontally). Each row corresponds to one genome. Genome keys are omitted for simplicity. Gray boxes indicate that no hits were found with default BLASTN parameters. (**B**) Example gene synteny comparisons of ICE*clc* with two recovered *P. aeruginosa* ICEs (ICE_19TCP and ICE_38987, extended list shown in Fig. S2). ORFs and their orientation are represented by blue boxes on top or bottom strand (reverse orientation). Known or genes with clear annotation are indicated (simplified per operon, e.g., *ars* for arsenic resistance operon). Bars highlight regions of homology among the three ICEs; darker hues indicating higher similarity (in BLASTN). Homology regions correspond to what is referred to as *core* of the ICEs. (**C**) Number of *P. aeruginosa* genomes with BLASTN hits above default thresholds to *intB13*, *traI*, *iceB4*, or *bisD* from ICE*clc* as proxies for the number of ICE*clc*-like elements per draft genome. (**D**) Multiple ICE*clc-*like elements per genome, shown here as regions with BLASTN similarities above default thresholds to ICE*clc* for genomes of *P. aeruginosa* strains 35969 and 17090.

First, we tried to obtain a broad overview of the presence of ICEs related to ICE*clc*. For this, we focused on 40 ICE*clc* genes, which we expected might be conserved: the *intB13* integrase, the *traI* relaxase, the DNA topoisomerase *topB*, 13 open reading frames (ORFs) whose products are involved in regulation and activation of the element (33), and 24 ORFs whose products are implicated in the type IV ICE conjugative system (37). BLASTN searches with these queries and a 70% sequence identity cut-off against the 181 *P*. *aeruginosa* draft genomes returned hits to *intB13* for 91% of the genomes (74%–91% nucleotide identity over 63%–94% of the gene length, Fig. 1A). Additionally, 92% of the isolates produced hits to *traI* (81%–85% nucleotide identity across 54%–97% of the gene length, Fig. 1A). Other ICE*clc* genes were, in contrast, less conserved among the *P. aeruginosa* genomes. For instance, among the regulation genes, we found hits for 91% of the genomes to *bisD*, but only 6% to *bisR* (Fig. 1A), and none of the isolates carried analogs of the ICE*clc* regulatory genes *mfsR*, *marR*, or *tciR* (47). A large proportion of the suspected ICE regions in the *P. aeruginosa* genomes carried homologs of *parA*. ParA-related proteins were previously found to be important for maintenance of the extrachromosomal circular form of the pathogenicity island PAPI-1 of *P. aeruginosa* PA14 (48) and causative of growth impairment linked to the presence of ICE*clc* in *Pseudomonas putida* (49). Finally, some genomes only carried a few ICE*clc*-related genes such as *inrR*, *iceD4*, and/or *orf55476* analogs but no other detectable ICE*clc* gene homologies (Fig. 1A). These may thus not consist of bona fide complete ICE regions but of spurious hits. These results suggested ICE*clc-*like elements to be widely distributed among the *P. aeruginosa* isolates.

To better delineate the continuity and gene content of the detected putative ICEs, we focused on a smaller set of sequences by excluding obvious redundancy. This was judged from similarity of a 200-kb region around the position of the identified *traI* gene(s) and the provenance of the strain (Table S1). If two or more 200-kb regions were the same between strains isolated from the same source, we assumed they originated from the same *P. aeruginosa* clone, and only one was taken for further analysis. This resulted in 85 regions with potential ICEs. Next, we manually searched for the ICE “boundaries,” consisting of regions carrying an expected 18-bp repeat sequence similar to that within the identified ICE*clc* attachment (*att*) sites (Table S2). We expected the *att* sites to be identical or closely related to the *att* sites previously identified in ICE*clc* as the newly identified putative ICEs share a closely related integrase to *intB13* (35). Eighteen ICEs had identical direct repeats as in ICE*clc* (e.g., ICE_13520; both *attR* and *attL* contain the 18-bp “GTCTCGTTTCCCGCTCCA”; Table S2), whereas 50 showed one nucleotide mismatch in the putative *attR* repeat (but with *attL* still being identical to ICE*clc*, e.g., ICE_13524; Table S2). The *att* repeat sequences of these ICEs correspond to the 3′-end 18 bp of the second, third, and fourth copies of the tRNA^Gly^ gene in the *dnaA*-aligned reference genome (Fig. S1). One element (ICE_17090) contained a different set of direct repeats and was integrated into the first copy of the five tRNA^Gly^ genes on the *P. aeruginosa* genome (Fig. S1). The remaining 16 ICE regions (e.g., ICE_27983 and ICE_34204) have contig gaps at their end or beginning and could not be delineated further to include the 18-bp repeat sequences; the assembly gaps were used as fictive boundaries, and we can only make a minimum conservative size estimate (Table S2). Assuming the 18-bp repeats or contig gaps as ICE boundaries, their sizes range between 57 and 133 kb (Table S2). All except two identified ICEs encode a single relaxase (TraI) belonging to the MOB_H_ family (Table S2) (50). The other two ICEs (ICE_38702 and ICE_36935) encode one relaxase of the MOB_H_ family and a second one belonging to the MOB_P_ family. BLASTN comparisons of the presumed *P. aeruginosa* ICEs against ICE*clc* indicated that the gene synteny of the conserved region is broadly maintained, and most of the variable genes are located in between the integrase gene and *traI* (examples shown in Fig. 1B; Fig. S2).

To better understand if individual hosts could carry multiple ICEs of the same or of different types, we selected four ICE*clc* genes (*intB13*, *traI*, *iceB4*, and *bisD*) and used the number of BLASTN hits as a proxy for the number of ICE*clc*-like ICEs per *P. aeruginosa* genome (Fig. 1C). Assuming that one set of these four genes corresponds to one element, most genomes carried either one or two ICE*clc*-type ICEs, while few had three or none at all (Fig. 1C). To obtain a broader picture on the presence of other ICE types than ICE*clc*, we scanned a single representative of each of 21 different *P. aeruginosa* “clones” (see below) for ICEs and integrative and mobilizable elements (IMEs) using ICEfinder (https://bioinfo-mml.sjtu.edu.cn/ICEfinder/ICEfinder.html). IMEs are non-self-transmissible elements; instead, they rely on the presence of a conjugative element to mediate their horizontal transfer (51). On average, each genome carried 2.8 ICEs (between 1 and 5) and 0.67 IMEs (between 0 and 2) (Table S3), of which one ICE*clc*-like element and others belonged to a different category. Four of the 21 genomes carried two distinct ICE*clc*-like elements (Table S3). This suggests that the number of ICE*clc*-like elements found by single gene comparisons might be an overestimate and resulting from hits to ICEs more distantly related to ICE*clc* (as in Fig. 1D). Some isolates were found to carry two ICE*clc*-type elements in tandem, as in the case of strain 35969 (Fig. 1D), whereas others contained ICE*clc-*like elements in different loci of their genome (strain 17090, Fig. 1D). In addition, these strains showed presence of a phylogenetically more distant ICE, of which only certain ICE*clc* gene homologs were picked up above the BLASTN thresholds (70% sequence identity, Fig. 1D). To find further similarity between the 85 putative ICEs and other known ICE-like elements, we compared them by BLASTN against the previously characterized *P. aeruginosa* genomic islands PAGI-2, PAGI-3, PAPI-1, and LESGI-3 (5, 14, 48). We then filtered the results to keep only the hits with a query coverage higher than 60%. This resulted in 53 elements having 84%–97% nucleotide identity to LESGI-3, limited to a region of 61%–80% of their total length (Table S2). Accordingly, 54 elements had 84%–93% identity to PAGI-2, limited to a region of 61%–83% of their total length, while ICE_36935 is 100% identical to PAGI-2 over its whole length (Table S2). None of the identified ICEs had extensive homology to PAGI-3 or PAPI-1 elements (maximum coverage detected by BLASTN: 23% and 6%, respectively, of total ICE length).

### Circulating ICE clones are phylogenetically congruent with their *P. aeruginosa* host

**Fig 2:**
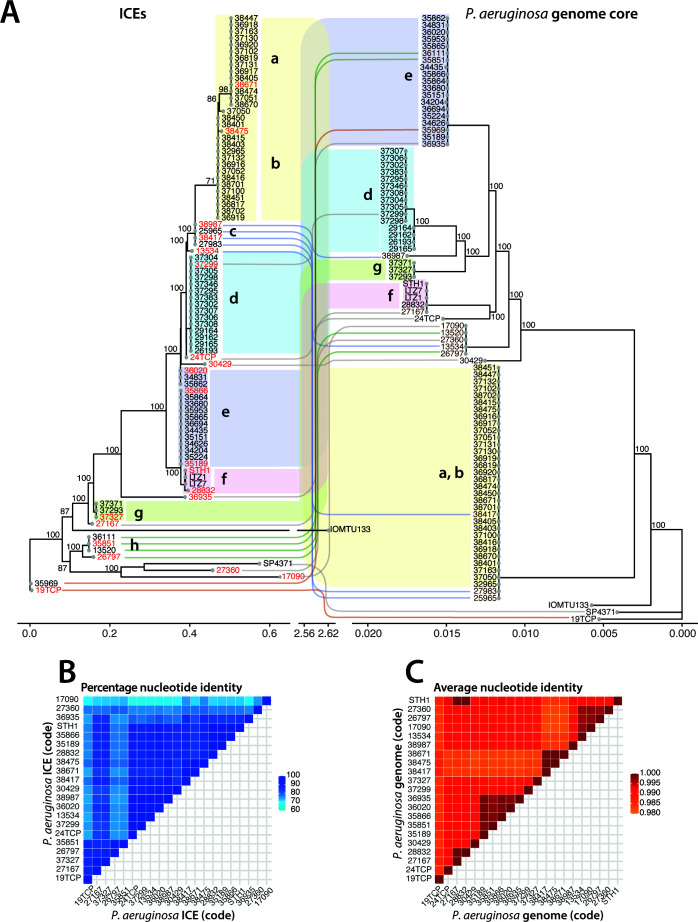
Phylogenetic comparative analysis of *P. aeruginosa* hosts and their ICE*clc* family members. (**A**) Bootstrapped (*n* = 1,000) consensus phylogenetic trees of 85 representative *P. aeruginosa* genomes and ICEs from the ICE*clc* family. Two *P. aeruginosa* strains and their ICEs were added as outgroups (IOMTU133 and SP4371 from reference 38). The host “genome core” tree (right) is based on the concatenated alignment of all shared single-copy orthologous genes. ICE tree is based on a multiple sequence alignment of complete sequences. Gray and colored connecting lines indicate a different position in the ICE and genome core trees between the left and right, while color-shaded regions and letters (a–g) connect common host-ICE lineages. ICE names in red indicate those selected for further analysis. *P. aeruginosa* host names in brown fonts indicate those genomes harboring an ICE different from expected from their sublineage. The scale bar represents the expected average number of nucleotide substitutions per site. The scale of the ICE tree is broken between 0.6 and 2.56 nucleotide substitutions per site for visualization of the outlier “IOMTU133.” (**B**) Pair-wise percentage nucleotide identities (as heatmap according to colored scale) among 21 representative ICEs (i.e., those labeled red in panel A) from sequence alignments over the complete deduced ICE region. (**C**) Pair-wise average nucleotide identities among the 21 corresponding genomes to panel **B** (i.e., those labeled red in panel A) calculated across 30-bp sliding windows.

To assess the phylogenetic relationship among the 85 representative ICEs and between their hosts, we compared maximum-likelihood phylogenetic trees of both. The ICE tree was constructed from a multiple sequence alignment on the complete sequence of each element and bootstrapped (*n* = 1,000) to find majority branches (Fig. 2A). As outgroups, we added ICEs from two *P. aeruginosa* strains selected from the study of Botelho et al. (38). These ICE*clc*-like elements encode carbapenemases (*bla*_DIM-1_ in IOMTU133, GenBank accession number GCA_001548335; and *bla*_NDM-1_ and *bla*_PME-1_ in SP4371, GenBank accession number GCA_003950255). The tree topology suggested there are eight major circulating clones (labeled *a–h* in Fig. 2A), plus several more distinct ICEs, with as low as 60% similarity (in terms of percentage of nucleotide identity, Fig. 2B). Six of the major ICE groups were found in similarly clustered host genome phylogeny, which was constructed from alignments of all the single-copy core orthologous genes (*n* = 4,260 genes; clusters a, b, d, e, f, and g; Fig. 2A). This suggests clonal divergence of those ICEs with their host genomes. The host phylogeny suggests that most of the isolates are clonal. Indeed, their nucleotide diversity is extremely low or inexistent (in terms of average nucleotide identity, Fig. 2C). In contrast, notably ICE groups c and h find themselves in different *P. aeruginosa* host lineages (a, b, and e, respectively; Fig. 2A), whereas additional distinct individual ICEs are found within clonal host lineages (as highlighted with brown labels and colored or gray lines in Fig. 2A). For example, *P. aeruginosa* strain 27167 is clonal to strains 28832, STH1, LTZ7, and LTZ1 (group f) but carries an ICE that is divergent from the ones carried by the other four strains (ICE group f). This strongly suggests that, in addition to clonal divergence, there is horizontal transfer of the ICEs in the hospital environment.

**Fig 3:**
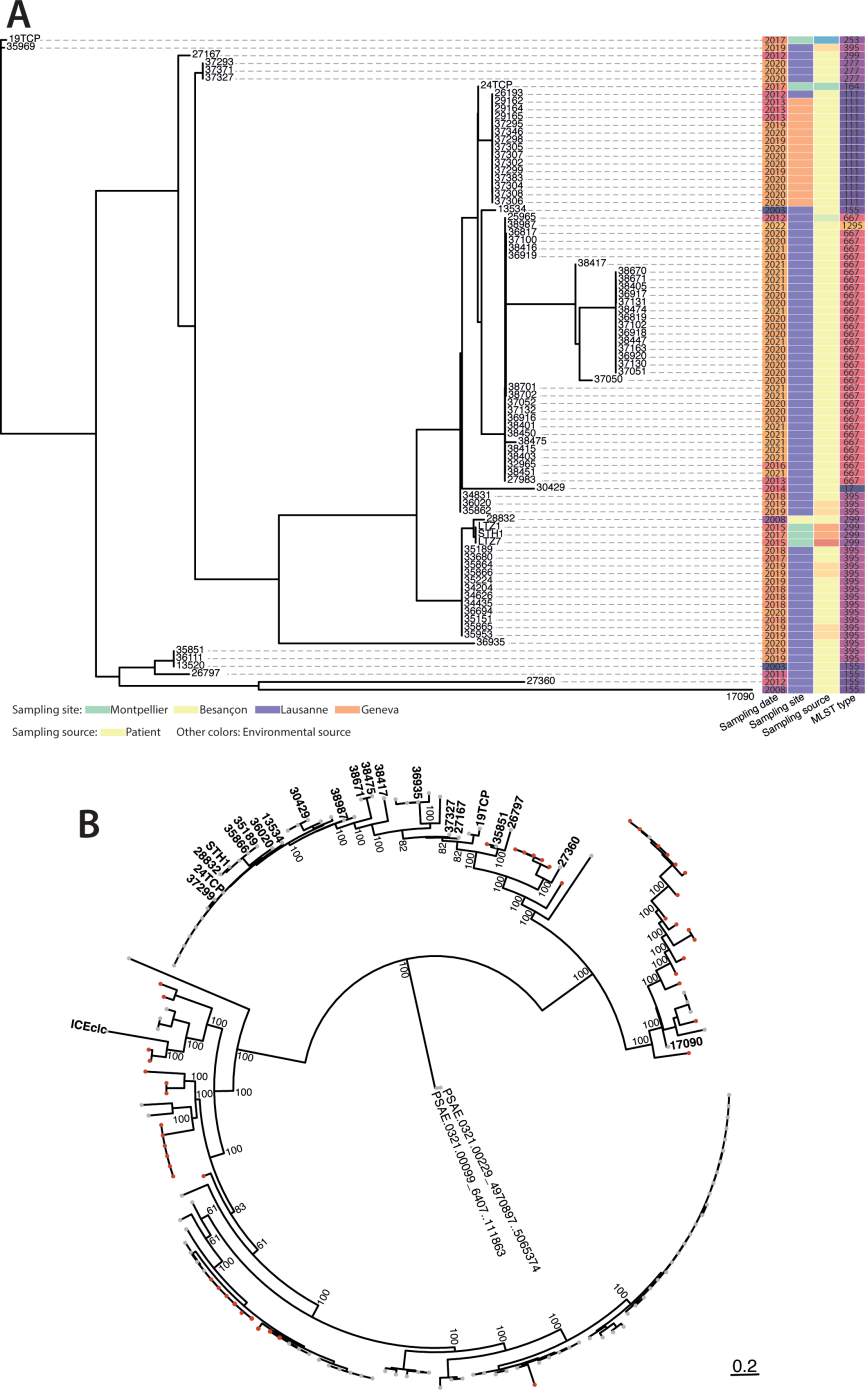
Comparative phylogenetics of *P. aeruginosa* ICE*clc* family elements. (**A**) Correlation of ICE phylogenetic signal with the date and location of strain sampling, isolation source (light yellow, patients; other colors, various environmental sources; see Table S1) and MLST of the host. The phylogenetic tree is the same as in Fig. 2A except that the outgroups are omitted. (**B**) Phylogenetic comparative analysis of publicly available *P. aeruginosa* ICE*clc* family members. The bootstrapped (*n* = 1,000) consensus phylogenetic tree includes 139 *P*. *aeruginosa* ICEs from the ICE*clc* family (from reference 44) and the 21 ICEs analyzed in this study. For simplicity, only the ICE code numbers (IDs) of the latter and ICE*clc* are indicated on the tree; details of the former are available in Table S6. As outgroups, we included two *P. aeruginosa* ICEs randomly sampled from the sequences that were not considered of the *clc* family (namely, ICEs with a hit above 70% sequence identity to less than 20 of the ICE*clc* core genes; IDs PSAE.0321.00229_4970897.5065374 and PSAE.0321.00099_6407.111863 in Table S14 of reference 44). The tree is based on a multiple sequence alignment of complete ICE sequences. Gray tips represent ICEs without detectable anti-microbial resistance, while red tips indicate ICEs carrying anti-microbial resistance determinants (from Table S14 of reference 44). The scale bar represents the expected average number of nucleotide substitutions per site.

We next correlated the ICE phylogenetic signal with metadata such as the date and geographical location of sampling, isolation source, and MLST of the host (Fig. 3A). Some of the ICEs found in strains isolated in the 2000s are still found in more recent strains. For example, ICE_28832 isolated in 2008 is closely related to ICE_STH1, which is present in a *P. aeruginosa* strain isolated in 2017. Some others, like ICE_13524, isolated in 2003, are absent in more recent isolates from our data set. Interestingly, a few closely related ICEs are found in strains isolated in different geographical regions (e.g., ICE_26193, from a strain isolated in 2012 in Lausanne, is closely related to ICE_37295, present in a *P. aeruginosa* isolated in 2020 in Geneva). The strains carrying these ICEs are closely related in their host phylogeny (both *P. aeruginosa* 26193 and 37295 belong to group d, Fig. 2A). This suggests that the same *P. aeruginosa* clones are circulating within multiple hospitals. Also, the same ICEs are found in isolates from different origins, such as infected patients or hospital environment, potentially implying that contaminated surfaces in the clinics are a reservoir of the strains that infect patients and vice versa (e.g., ICE_34831, isolated from an infected patient, is closely related to ICE_36020 and ICE_35862, isolated from sink traps).

### Micro-evolution of genes within the *P. aeruginosa* ICE conserved core

From the broad phylogeny of ICEs, we selected one element from each branch (red labels in Fig. 2A) to analyze conservation and micro-variations in their core genes. This resulted in 21 elements, which are a non-redundant representation of the diversity of circulating ICEs in the four hospitals. To place this local ICE diversity in perspective with worldwide circulating elements, we extracted 139 *clc*-like ICE sequences identified among 2009 publicly available and pruned *P. aeruginosa* genomes (44). We constructed a bootstrapped (*n* = 1,000) maximum-likelihood phylogenetic tree rooted with two outgroups that were randomly sampled from the same data set (44) but were not considered ICEs of the *clc* type (Fig. 3B). The ICE*clc*-type tree is composed of two to three major clades, one of which is broadly covered by most of the local ICEs (Fig. 3B). This indicates further ICE*clc-*type clonal diversification exists, which may become relevant in the context of anti-microbial resistance determinants (Fig. 3B, red symbols; see below).

**Fig 4:**
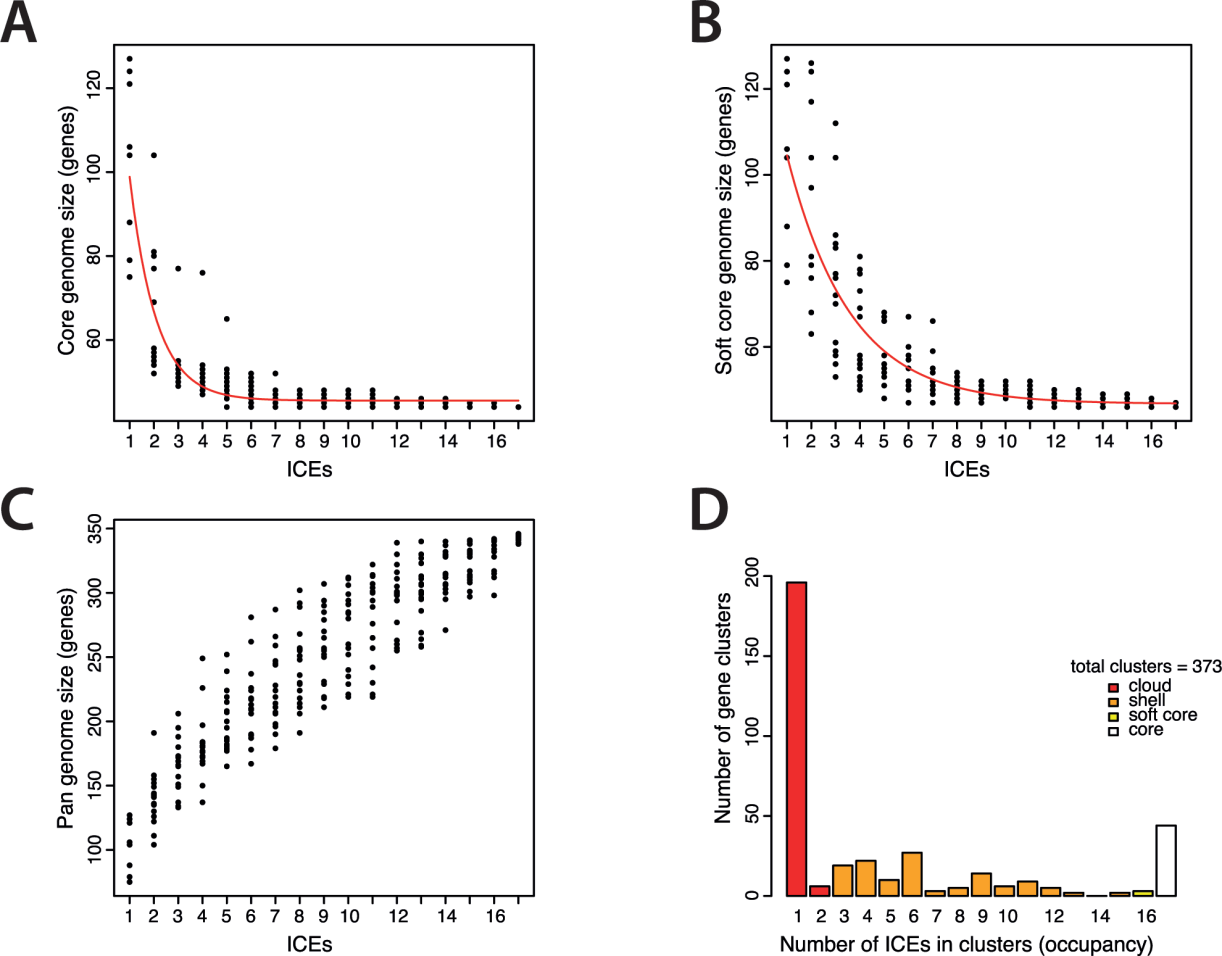
Core- and pan-genome sizes of *P. aeruginosa* ICEs of the (local) ICE*clc* family. (**A**) Estimate of the number of genes making up the ICE core (*n* = 17 complete ICEs included). ICEs with assembly gaps (*n* = 6 ICEs) were omitted for a better estimation of true core-genome sizes. Red line represents the Tettelin fit (52). Each dot represents 1 of 18 independent subsampling of *n* = 1–17 ICEs. (**B**) Estimate of the number of genes making up the ICE soft core (i.e., the genes shared by 95% of the elements). Red line represents the Tettelin fit. (**C**) Estimate of the ICE pan-genome size (cutoff for similarity: maximum *e* value of BLAST comparisons = 0.00001 and minimum coverage length = 75%). (**D**) Attribution of the ICE genes into cloud (i.e., number of genes present in 1–2 of 17 ICEs), shell (i.e., number of genes present in 3–15 of 17 ICEs), soft-core (i.e., number of genes present in 16 of 17 ICEs), and core (i.e., number of genes present in 17 of 17 ICEs) compartments.

We inferred the core- and pan-genome sizes (Fig. 4A through D) considering only the elements without assembly gaps (16 out of 21) and including ICE*clc*. The size of the core ICE genome, defined as the set of orthologous genes present in every element, saturates quickly when increasing the number of elements taken into consideration and tends toward 44 genes (Fig. 4A). The size of the soft core, defined as the set of genes present in 95% of the elements, shows a similar behavior but approaches 47 genes (Fig. 4B). The pan-genome size continually grows as more elements are added and does not seem to reach a saturation with the 17 selected ICEs (here at 350 genes, Fig. 4C), suggesting that new and unique variable genes are present in all the different elements. Accordingly, the cloud ICE genome, defined as the genes present only in one to two elements, is the most abundant class (Fig. 4D).

**Fig 5:**
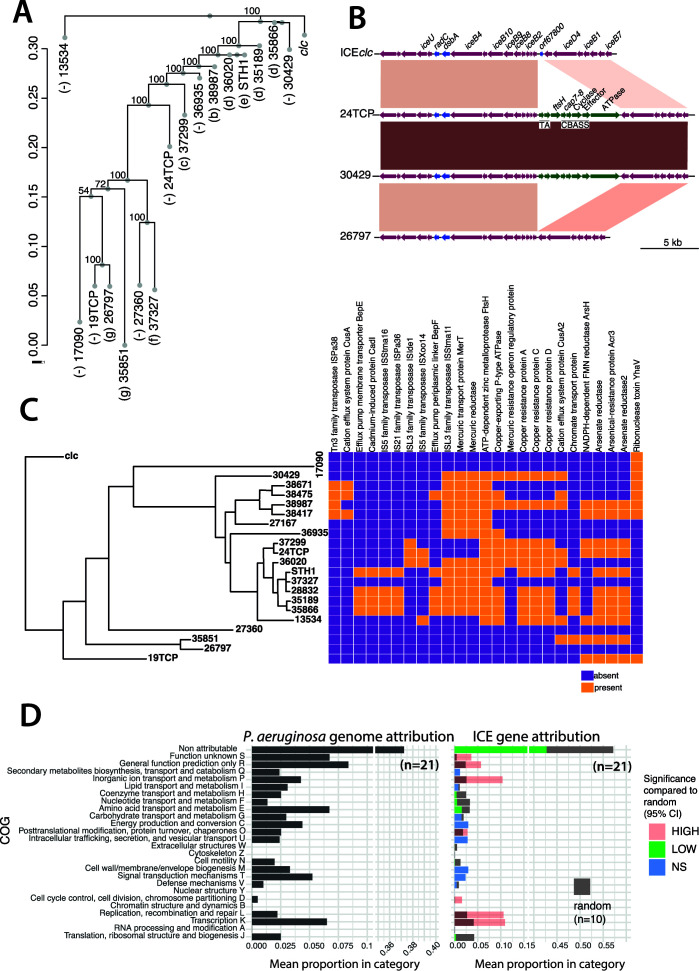
Evolution of ICE*clc* family core and variable regions. (**A**) Consensus bootstrapped (*n* = 1,000) ICE phylogenetic tree based on established core genes (*n* = 42, single-copy orthologous genes) for the 16 complete ICEs selected from Fig. 2A (in red) with ICE*clc* as outgroup. Letters in brackets correspond to the subgroups of Fig. 2A. (**B**) Micro-divergence within the core conjugative gene region. Gene maps of the ICE*clc* conjugative locus versus three *P. aeruginosa* ICEs (ICE_24TCP, ICE_30429, and ICE_26797). ORFs and their orientation are represented by arrows; blue arrows indicate non-core genes; magenta arrows indicate core genes, and green arrows indicate gene insertions in two of the *P. aeruginosa* ICEs. Connecting colored blocks represent BLASTN similarities (darker colors indicate higher similarities). Gene annotation is based on published data (37) or BLASTP homologies. (**C**) Gene gain and losses (subset) across the 21 selected ICEs in conjunction with their phylogenetic positioning (consensus bootstrapped tree based on a multiple sequence alignment of complete sequences). (**D**) Enrichment or depletion in attributed functional classes of ICE-located genes in comparison to the *P. aeruginosa* genomes. Bars show mean proportions of cluster of orthologous groups (as indicated) class attribution from the annotated genes of the 21 *P*. *aeruginosa* genomes (ICE regions included) and their corresponding selected ICEs. Colors in the right panel indicate classes for which the observed mean proportion is bigger (high, red), smaller (low, green), or not significantly (blue) different from the 95% CI around the mean proportion attributed from 10 randomly sampled subsets of single-copy pan-genomic (all 21 *P*. *aeruginosa* isolates) chromosomal genes, which are shown as gray bars in the right panel. CBASS, cyclic-oligonucleotide-based anti-phage signaling system; TA, toxin-anti-toxin system.

To address the extent of conservation in the defined core genome, we constructed a bootstrapped (*n* = 1,000) maximum-likelihood phylogenetic tree of the ICE core, inferred from all the single-copy orthologous core genes (*n* = 42 genes) (Fig. 5A). This list of core orthologous genes encompasses those that were experimentally confirmed for the ICE*clc* element and globally speaking codes for functions that control the lifestyle and transmission of the elements (Table S4). The core gene phylogeny mostly confirmed the tree topology of the dominant circulating ICEs based on their complete sequence (see indicated group names from Fig. 2A within names of Fig. 5A) but more clearly shows the divergence of the less common ICEs.

At the level of individual conserved but non-core genes, the percentage of nucleotide identity was as low as 40%, as shown, e.g., for the integrase genes (Fig. S3). However, the integrase comparison further showed that ICE lifestyle modules comprise gene mosaics rather than strict coherent co-evolving entities. For example, the integrase tree has only 10 different integrase “branches” among the 21 groups (Fig. S3), and five putative ICEs carry two distinct integrases (magenta in Fig. S3) with ~70% nucleotide identity. Also, detailed inspection of gene order within core regions indicates that there are specific gene insertions and losses (Fig. S2). For example, we found an insertion of defense system genes within the conjugative locus of, e.g., ICE_24TCP and ICE_30429 (Fig. 5B, green arrows), with deletion of the ICE*clc* gene *orf67800*. This acquired locus contains a toxin-anti-toxin system (‘TA” in Fig. 5B) composed of a DNA-binding protein (anti-toxin) and a PIN domain containing protein (toxin). Additionally, it encodes a cyclic-oligonucleotide-based anti-phage signaling system, a defense system involving cell death prior to phage replication (53, 54). This system is composed of an oligonucleotide cyclase gene of the CD-NTase family (“cyclase” in Fig. 5B), an “effector” (endonuclease) gene, and additional associated Cap7- and Cap8-encoding genes. This locus further encodes a zinc metalloprotease (FtsH) and an ATPase of the AAA family. This suggests, from an evolutionary point of view, that the ICE*clc*-like elements in *P. aeruginosa* indeed carry a “core,” which undergoes clonal mutational drift and is subject to small indels, and a variable region that allows larger-scale gene insertions and deletions.

### Variable region content indicates a wide variety of potentially adaptive genes among *P. aeruginosa* ICE*clc*-like elements

Since one would expect that the genes outside the immediate ICE lifestyle and transmission functions are potentially providing adaptive benefits to the host strains, we next analyzed in more detail the types of variable genes and their occurrences within the various predicted *att* borders of the ICEs. This interpretation is complicated by the large majority of hypothetical functions (i.e., without clear annotation and existing homologies; almost 75% of the genes, Table S5). However, a strikingly large abundance of predicted heavy metal resistance operons was detected among ICE variable regions (arsenic, cadmium, cobalt, copper, lead, mercury, nickel, and zinc; Table S5), with different patterns of co-occurrences among ICEs (Fig. 5C). For example, mercury resistance co-occurred with copper resistance, and in other cases with arsenic, or with cadmium resistance (Fig. 5C). In addition, we found a large fraction of annotated genes referred to (non-metal ion) efflux systems (e.g., BepE and BepF; enterobactin exporter EntS; toluene efflux pump TtgI; and multidrug resistance proteins MdtA, MdtE, and MexB) or transport energizing systems (e.g., potassium-transporting ATPase, 59 total occurrences in 85 ICEs), which may contribute to the capacity of the *P. aeruginosa* hosts to withstand antibiotic or anti-microbial compounds (55–59). Finally, some functions circulating in the ICE variable regions may point to adaptations to environmental or human-host conditions (Table S5). For example, a common ATP-dependent zinc metalloprotease (75 total occurrences in 85 ICEs) has been associated with virulence and survival of *P. aeruginosa* (60, 61). Other frequent, commonly found variable genes included a gene for polyphosphate ADP phosphotransferase, which may increase the capacity of the cell to scavenge phosphate; a fatty acid methyltransferase that could be involved in biotin synthesis; and quinone oxidoreductase 2, which associates to tricarboxylic acid (TCA) cycle components and 10-formyltetrahydrofolate synthesis (Table S5). Notably, local ICEs were remarkably depleted for clear anti-microbial resistance genes, which, however, are found in ICEs of the same major clade and the two other clades of the worldwide survey (Fig. 3B) (44).

In order to understand if ICEs are enriched for particular functional groups, which may be an indication of their selection, we compared the Cluster of Orthologous Groups (COG) distribution between the ICEs and their host genomes (Fig. 5D). We reasoned that if the cargo genes of the ICEs were under selection, their COG class distribution would be enriched compared to a random subselection of chromosomal genes. To test this hypothesis, we randomly sampled 106 genes (corresponding to the average number of genes present on the 21 analyzed ICEs) 10 times independently from the pool of all available chromosomal genes, classified them into COGs, and compared their average distribution and occurrence to those of the genes of the ICEs themselves (Fig. 5D). This showed that 11 out of the 25 COG categories are significantly underrepresented or overrepresented on the ICEs compared to the frequencies observed with randomly sampled chromosomal genes (Fig. 5D). For example, the class “inorganic ion transport and metabolism” is significantly enriched on the ICEs compared to the random sampling, which is in agreement with the noted abundance of heavy metal resistance operons on the elements. Also, genes in the COG classes “replication, recombination, and repair,” “transcription,” “cell cycle control,” “posttranslational modification,” and “general function prediction/unknown” were more represented than expected by chance (Fig. 5D). This suggests that these categories are under selection (and counterselection) on the local ICEs and may contribute to *P. aeruginosa* adaptation and survival under the Swiss hospital conditions.

## DISCUSSION

The temporal tracing of a single focal species group in a consistent environment by full-genome sequencing is a great opportunity to study ongoing micro-evolution of mobile genetic elements such as ICEs. Genome mining and comparative genomic analysis of 181 *P*. *aeruginosa* isolates from hospital environments sampled over 20 years and a wider comparison to publicly available pruned *P. aeruginosa* genomes revealed a large diversity and abundance of ICEs with similarity to ICE*clc* of *P. knackmussii* B13 (25). This indicated and confirmed previous results from other hospital environments that ICE*clc* family elements are pervasive among *P. aeruginosa* strains (5, 12, 44). Our phylogenetic analysis showed that circulating local *P. aeruginosa* clones have low nucleotide diversity among their chromosomal core regions but primarily diversify by their accessory genomic segments. More than 90% of the analyzed genomes carried one or multiple ICE*clc*-like ICEs and frequently contained also ICEs or IMEs from other families, similarly as to recent conclusions from analysis of publicly available complete *P. aeruginosa* genomes (44). The ICE*clc* family members are characterized by a conserved core region that encodes basic lifestyle functions, such as the conjugative machinery, but they are diversified through different variable gene contents.

Phylogenetic comparison of the local *P. aeruginosa* chromosomal and ICE orthologous genes indicated both clonal ICE evolution as well as “non-matching” pairs. This is strong evidence that the ICEs are transferred horizontally among the different *P. aeruginosa* isolates in the hospital environment. Highly similar ICEs were also found in strains isolated from the other three hospitals, suggesting that both exchange of strains and of their ICEs can occur and contribute to *P. aeruginosa* diversification and adaptation. As far as we could analyze, the ICE*clc* family members appear to encode all genes necessary for their horizontal gene transfer, in comparison to a recent functional study of ICE*clc* conjugative transfer genes (37). This includes all genes for a complete type IV conjugative system, an integrase, and a relaxase gene. Some ICEs (e.g., ICE_38702 and ICE_36935) encoded two relaxases of different families, which may have consequences not only on the transfer dynamics of these ICEs themselves but also potentially on other co-mobilizable elements (IMEs) in *P. aeruginosa*.

In contrast, the characterized regulatory elements for ICE*clc* activation were only partly conserved among the *P. aeruginosa* ICE*clc* members. This suggests that control of ICE activation in *P. aeruginosa* may be distinct from that of ICE*clc* in *P. knackmussii* B13 and may respond to different environmental cues (ICE*clc* is activated in stationary-phase cells after growth on 3-chlorobenzoate). For instance, no homologs were detected among the *P. aeruginosa* ICEs for the *mfsR* and *tciR* genes, which are thought to provide the first level of control on ICE*clc* activation in *P. knackmussii* and *P. putida* during exponential growth (47). This confirms our previous assumption that (notably) insertion of the *mfsR* regulatory gene and the corresponding multidrug efflux system were an evolutionary novelty on ICE*clc* (32). In addition, only few *P. aeruginosa* ICE*clc* members encoded close homologs of *alpA* and *bisR*, which form the second regulatory layer of ICE*clc* activation (33)(47). Interestingly, however, the ICE*clc bisDC* regulatory genes responsible for formation of bistability and control of downstream ICE genes (such as the integrase and the conjugation system) were again conserved among all local *P. aeruginosa* ICE*clc* family members. This would indicate that the eliciting environmental or physiological cues influencing *P. aeruginosa* ICE activation are different from ICE*clc*, but once activated, these ICEs would also show bistable phenotypes of transfer-competent and “ICE-silent” cells (62). So far, however, the frequency of activation and ICE transfer from the *P. aeruginosa* hospital isolates has not been measured.

Although all local *P. aeruginosa* ICE*clc* members encompassed a conserved set of ~40 orthologous genes, the corresponding gene modules and gene syntenies showed evidence of mosaic structure. This indicates that they are not strictly coherent co-evolving entities but can undergo further gene insertions and deletions, as well as mutational drift. This may occur, for instance, by recombination between modular cores of different ICEs within the same host, which may also lead to the acquisition of new genes or regulatory elements and can contribute to the evolution of novel ICE variants. From an evolutionary point of view, this suggests that the core of the ICEs plays a critical role in their survival and persistence over time. In contrast, the permissiveness for gene insertions and deletions in their “variable” regions allows for even greater flexibility and adaptability and permits larger-scale changes that may result in the acquisition or loss of functional genes that confer a selective advantage to the host organism.

Not surprisingly, the uncovered *P. aeruginosa* ICE*clc* members carried a wide range of variable gene functions. Notably, genes not only for heavy metal tolerance but also for regulatory elements were commonly present and enriched with respect to random models, suggesting they are under positive selection. Accordingly, it seemed that, over time, the ICEs are acquiring more heavy metal resistance determinants, as these were less abundant in ICEs found in strains isolated earlier in time (Fig. 3A and 5C). Other ICEs have been detected in *P. aeruginosa* that encode heavy metal resistance, such as PAGI-2. Previously, a 99.972% ICE similar to PAGI-2 was found in *Ralstonia metallidurans* CH34 (5), which was described to grow in millimolar concentrations of toxic heavy metals. This suggests by analogy that the heavy metal resistance genes on the ICEs in the *P. aeruginosa* clones can contribute to their tolerance against heavy metal stress. Why this remains so strongly conserved and pervasive among the Lausanne/Geneva hospital clones is unclear. Potentially, their positive selection is related to the presence of metallic copper coating in the water systems of healthcare institutions, which are known to be frequently colonized by copper-tolerant *P. aeruginosa* and to act as a reservoir of opportunistic strains (63–66). Additionally, there may be hidden mechanisms of cross-resistance between heavy metals and other anti-microbial compounds, such as those described for copper and antibiotic cross-resistance (64).

Even though we did not detect any specific antibiotic-resistance genes among the local *P. aeruginosa* ICE*clc* members, notably, genes for carbapenem resistance have been acquired by ICE*clc*-like elements in other *P. aeruginosa* hospital strains, e.g., in Portugal and South Korea (38, 42). Furthermore, comparison to a worldwide survey of *P. aeruginosa* genomes carrying ICE*clc*-type elements that were identified in a 2023 study by Botelho and coworkers (44) showed that many related elements are present among the three major ICE*clc* clades with genes for anti-microbial compound resistance (Fig. 3B). It may thus just be a matter of time and selection pressure before such genes, either by recombination onto existing ICEs, by conjugative transfer of the corresponding ICEs, or by transmission of the *P. aeruginosa* host, find their way into the local hospital environment. Further inconspicuous potential resistance or virulence factors may be present on the local ICEs described here. These include a number of efflux systems which are associated with increased tolerance to anti-microbials, and an ATP-dependent Zinc metalloprotease FtsH, homologs of which have been involved in a variety of phenotypes associated to virulence and survival (60, 61). In conclusion, our findings support the importance of adaptive mobile genetic elements in the success of *P. aeruginosa* as an opportunistic pathogen.

## MATERIALS AND METHODS

### Strain isolations and sequencing

Strains from the CHUV were isolated and typed during ongoing infection control surveillance and investigations, some of which have been described elsewhere (67–69). Strains from CHU Besançon originated from a study on the evaluation of a new typing system (70). Strains from HUG were isolated during an outbreak of VIM-producing *P. aeruginosa* (71). Strains from the CHU Montpellier were isolated in the course of a research protocol (ClinicalTrials.gov ID: NCT02751658) that studied the dynamics of hospital environment contamination by waterborne pathogens associated to healthcare infections. All strains were sequenced using Illumina MiSeq technology.

### Quality check and *de novo* assembly

The quality of the Illumina raw reads was assessed with FastQC (v.0.11.9) (72). Reads were trimmed with *trimmomatic* (v.0.39) (73) to remove short and low-quality reads and adapters. Genomes were *de novo* assembled with SPAdes (v.3.15.2) (74). The resulting contigs were filtered to keep only those longer than 500 bp and with a kmer coverage higher than 10. Single chromosomes were generated with RagTag (v.2.1.0) (75) from the filtered contigs of every genome by scaffolding and patching the filtered contigs against a reference complete genome of *P. aeruginosa* strain H26027 (NCBI accession number CP033684), also isolated from the Lausanne University Hospital. The genome scaffolds have been deposited with the European Nucleotide Archives under the accession study number PRJEB61470.

The sequence type of each strain was determined using MLST (v.2.19.0) (76), a program which extracts seven single-copy housekeeping genes (*acsA*, *aroE*, *guaA*, *mutL*, *nuoD*, *ppsA*, and *trpE*) and compares their sequence identity to previously deposited allele combinations in the *P. aeruginosa* PubMLST database (77).

### Isolation, annotation, and comparison of ICE sequences

Forty ICE*clc* genes were used as query for BLASTN (v.2.13.0) (78) searches against the filtered contigs of each genome (using command line default parameters). To delineate the ICE*clc*-like elements from their draft *P. aeruginosa* genomes, we first defined regions of 100 kb upstream and downstream of the located *traI*-homolog using SeqKit (v.2.2.0) (79). Within these regions, potential ICE attachment sites were manually searched by homology to the ICE*clc* direct repeats (5′-GTCTCGTTTCCCGCTCCA-3′), one of which should be located at the end of a gene for *tRNA^Gly^* and the other located within 50–130 kb from the first, allowing up to two mismatches (35, 80). Delineated “boundary”-defined ICEs were then annotated with PROKKA (v.1.14.6) (81), which was also used to annotate the rest of the draft genome. Sequence comparison maps (Fig. 1B and 4B; Fig. S2) were generated using genoplotR (82).

The presence of a tRNA*-*encoding gene at the site of insertion was detected with tRNAscan-SE (v.2.0) (83). Relaxase families were determined using MOBscan (84). Pair-wise average nucleotide identities were calculated using fastANI (v.1.33) (85) for the genomes and Bio3d (v.2.4–3) (86) for the aligned ICEs and integrases. Percentages of GC nucleotide content were determined employing a sliding window method (https://github.com/DamienFr/GC_content_in_sliding_window) by using default parameters for the genomes, a window size of 100 bp and a step size of 10 for the ICEs. Estimations of the total number and variety of ICEs and IMEs were made by using ICEfinder (https://bioinfo-mml.sjtu.edu.cn/ICEfinder/ICEfinder.html) (87).

To have a broader overview on globally circulating ICE*clc*-type elements, we downloaded all the *P. aeruginosa* genomes based on Table S13 of reference (44) (a total of 203 genomes, accession numbers in Table S6). Suspected ICE sequences were extracted from their respective genomes based on the coordinates given in Table S13 of reference (44) using seqkit subseq (v.2.2.0) (79). The 477 resulting ICEs were blasted against the 40 identified ICE*clc* core genes. Only the ICEs with a hit above 70% sequence identity to at least 20 of the ICE*clc* core genes were considered *clc*-like and kept for further analysis, resulting in 139 ICEs (Table S6 and presented in Fig. 3B).

### Pan-genome and phylogenetic analysis

We calculated a pan-genome of all local ICEs (i.e., without including chromosomal genes), with the GET_HOMOLOGUES package (v.22082022) (88), under the following non-default parameters: the number of input sequences used for the generation of pan/core genomes was set to 18; and OrthoMCL (v1.4) (89) was used as clustering algorithm. Core and pan-genomes were computed with the compare_clusters.pl and parse_pangenome_matrix.pl scripts; rarefaction curves were generated using the plot_pancore_matrix.pl script (88).

For the comparative phylogenetic analysis of draft genomes of local *P. aeruginosa* clones and ICEs related to ICE*clc*, we concentrated on a set of 85 ICEs out of the initial set of 181 putative ICE regions. ICE*clc* family members were considered redundant if the 200-kb regions around the identified *traI* relaxase were similar among strains isolated from the same patient. To this set, we included ICEs from two external *P. aeruginosa* strains (NCBI accession numbers NZ_AP017302 and NZ_CP034369) from the study of Botelho et al. (38) that were found to encode carbapenem resistance genes. To build the species tree, with help of OrthoFinder (v.2.3.8) (90), we identified all single-copy orthologous genes among the 85 *P*. *aeruginosa* genomes and extracted all the corresponding nucleotide sequences using a custom python script, yielding a total of 4,260 ortholog genes. Next, the gene sequences were aligned with MAFFT (v.7.475) (91) and concatenated, from which a maximum-likelihood phylogenetic tree was built with IQ-TREE 2 (v.2.0.6) (92). The general time-reversible model, with unequal rates and unequal base frequencies, was used to compute the species tree (93). Trees were replicated in 1,000 bootstrappings to test the reliability of each branching. For the ICE*clc* family member and integrase trees, a multiple sequence alignment on the complete ICE or integrase sequence was produced with MAFFT, and the complete alignment was used to build the tree with IQ-TREE 2. For the ICE*clc* family member tree (Fig. 2A), the transversion model, with AG = CT and unequal base frequencies, was used to compute the tree. For the integrase tree (Fig. S3), the unequal transition/transversion rates and unequal base frequencies model were used to compute the tree (94). The tree of publicly available *P. aeruginosa* ICE*clc* family members (Fig. 3B) was computed using the transition model, with AC = GT, AT = CG, and unequal base frequencies. The ICE single-copy orthologous genes tree (Fig. 5A) was built with the same approach as the species tree [using the general time-reversible model of reference (93)]. Phylogenetic trees were displayed and compared with GGTREE (95) or dendextend (96).

Hierarchical orthologous groups were inferred using OMA standalone (v.2.5.0) (97), and the resulting phylogenetic profiles (Fig. 3A and 5C) were visualized using Phandango (98).

### COG analysis

COG classes encoded by ICEs and *P. aeruginosa* genomes were attributed using WebMGA (99). To estimate the enrichment for COG attributable functions among the ICEs, we compared the actual COG class distributions to those from a randomly sampled distribution model. The model was produced by random sampling of 106 genes (the average number of genes in the 21 ICEs) from a “virtual pan-genome,” repeated 10 times independently. The virtual pan-genome was a list with every gene appearing in the annotation of the 21 host genomes (except for the ICE regions), filled up to 6,295 total genes (the average number of genes in the 21 analyzed genomes) with “hypothetical proteins.” The subsampled gene lists were again attributed to their COG class, and the average per-class distribution was calculated over the 10 random samples. Significant differences among actual and expected distributions (in Fig. 5D) are those in which the actual average class proportion is bigger or smaller than the 95% CI around the average of the corresponding randomly drawn class proportions.

## Data Availability

The data sets generated and analyzed during the current study are available in the European Nucleotide Archives repository under the accession study number PRJEB61470.

## SUPPLEMENTAL MATERIAL

Fig. S1 msphere.00517-23-s0001.pdf Chromosome map of *P. aeruginosa* PAO1.

Fig. S2 msphere.00517-23-s0002.pdf Gene synteny comparisons among the 21 *P. aeruginosa* ICE*clc*-family elements.

Fig. S3 msphere.00517-23-s0003.pdf ICE relatedness inferred from similarities among their integrases.

Captions msphere.00517-23-s0004.docx Captions to all supplemental material.

Table S1 msphere.00517-23-s0005.xlsx Characteristics of *P. aeruginosa* isolates.

Table S2 msphere.00517-23-s0006.xlsx Characteristics of the attributed ICE*clc* family elements in individual *P. aeruginosa* isolates from the regional hospital.

Table S3 msphere.00517-23-s0007.xlsx Total attribution of integrative and conjugative/mobilizable elements in *P. aeruginosa* isolates from the regional hospital.

Table S4 msphere.00517-23-s0008.xlsx Deduced core functions of ICE*clc* family members.

Table S5 msphere.00517-23-s0009.xlsx Gene function occurrences among the ICE*clc* family gene "cloud" in regional *P. aeruginosa* clinical isolates.

Table S6 msphere.00517-23-s0010.xlsx ICE*clc* family members identified among a worldwide survey of publicly available *P. aeruginosa* genomes.

ASM does not own the copyrights to Supplemental Material that may be linked to, or accessed through, an article. The authors have granted ASM a non-exclusive, world-wide license to publish the Supplemental Material files. Please contact the corresponding author directly for reuse.

## References

[1] Ramos J-L. 2004. Genomics, life style and molecular architecture. In Pseudomonas. Springer, Boston, MA. doi:10.1007/978-1-4419-9086-0

[2] Greenwald MA, Wolfgang MC. 2022. The changing landscape of the cystic fibrosis lung environment: from the perspective of Pseudomonas aeruginosa. Curr Opin Pharmacol 65:102262. doi:10.1016/j.coph.2022.102262 35792519 PMC12312682

[3] Trautmann M, Lepper PM, Haller M. 2005. Ecology of Pseudomonas aeruginosa in the intensive care unit and the evolving role of water outlets as a reservoir of the organism. Am J Infect Control 33:S41–9. doi:10.1016/j.ajic.2005.03.006 15940115

[4] Stover CK, Pham XQ, Erwin AL, Mizoguchi SD, Warrener P, Hickey MJ, Brinkman FS, Hufnagle WO, Kowalik DJ, Lagrou M, et al.. 2000. Complete genome sequence of Pseudomonas aeruginosa PAO1, an opportunistic pathogen. Nature 406:959–964. doi:10.1038/35023079 10984043

[5] Larbig KD, Christmann A, Johann A, Klockgether J, Hartsch T, Merkl R, Wiehlmann L, Fritz H-J, Tümmler B. 2002. Gene islands integrated into tRNA ^Gly^ genes confer genome diversity on a Pseudomonas aeruginosa clone. J Bacteriol 184:6665–6680. doi:10.1128/JB.184.23.6665-6680.2002 12426355 PMC135438

[6] Wolfgang MC, Kulasekara BR, Liang X, Boyd D, Wu K, Yang Q, Miyada CG, Lory S. 2003. Conservation of genome content and virulence determinants among clinical and environmental isolates of Pseudomonas aeruginosa. Proc Natl Acad Sci U S A 100:8484–8489. doi:10.1073/pnas.0832438100 12815109 PMC166255

[7] Klockgether J, Reva O, Larbig K, Tümmler B. 2004. Sequence analysis of the mobile genome island pKLC102 of Pseudomonas aeruginosa C. J Bacteriol 186:518–534. doi:10.1128/JB.186.2.518-534.2004 14702321 PMC305764

[8] He J, Baldini RL, Déziel E, Saucier M, Zhang Q, Liberati NT, Lee D, Urbach J, Goodman HM, Rahme LG. 2004. The broad host range pathogen Pseudomonas aeruginosa strain PA14 carries two pathogenicity islands harboring plant and animal virulence genes. Proc Natl Acad Sci U S A 101:2530–2535. doi:10.1073/pnas.0304622101 14983043 PMC356984

[9] Spencer DH, Kas A, Smith EE, Raymond CK, Sims EH, Hastings M, Burns JL, Kaul R, Olson MV. 2003. Whole-genome sequence variation among multiple isolates of Pseudomonas aeruginosa. J Bacteriol 185:1316–1325. doi:10.1128/JB.185.4.1316-1325.2003 12562802 PMC142842

[10] Lee DG, Urbach JM, Wu G, Liberati NT, Feinbaum RL, Miyata S, Diggins LT, He J, Saucier M, Déziel E, Friedman L, Li L, Grills G, Montgomery K, Kucherlapati R, Rahme LG, Ausubel FM. 2006. Genomic analysis reveals that Pseudomonas aeruginosa virulence is combinatorial. Genome Biol 7:R90. doi:10.1186/gb-2006-7-10-r90 17038190 PMC1794565

[11] Cramer N, Klockgether J, Wrasman K, Schmidt M, Davenport CF, Tümmler B. 2011. Microevolution of the major common Pseudomonas aeruginosa clones C and PA14 in cystic fibrosis lungs. Environ Microbiol 13:1690–1704. doi:10.1111/j.1462-2920.2011.02483.x 21492363

[12] Klockgether J, Würdemann D, Reva O, Wiehlmann L, Tümmler B. 2007. Diversity of the abundant pKLC102/PAGI-2 family of genomic islands in Pseudomonas aeruginosa. J Bacteriol 189:2443–2459. doi:10.1128/JB.01688-06 17194795 PMC1899365

[13] Wiehlmann L, Wagner G, Cramer N, Siebert B, Gudowius P, Morales G, Köhler T, van Delden C, Weinel C, Slickers P, Tümmler B. 2007. Population structure of Pseudomonas aeruginosa. Proc Natl Acad Sci U S A 104:8101–8106. doi:10.1073/pnas.0609213104 17468398 PMC1876578

[14] Kung VL, Ozer EA, Hauser AR. 2010. The accessory genome of Pseudomonas aeruginosa. Microbiol Mol Biol Rev 74:621–641. doi:10.1128/MMBR.00027-10 21119020 PMC3008168

[15] Mathee K, Narasimhan G, Valdes C, Qiu X, Matewish JM, Koehrsen M, Rokas A, Yandava CN, Engels R, Zeng E, Olavarietta R, Doud M, Smith RS, Montgomery P, White JR, Godfrey PA, Kodira C, Birren B, Galagan JE, Lory S. 2008. Dynamics of Pseudomonas aeruginosa genome evolution. Proc Natl Acad Sci U S A 105:3100–3105. doi:10.1073/pnas.0711982105 18287045 PMC2268591

[16] Guglielmini J, Quintais L, Garcillán-Barcia MP, de la Cruz F, Rocha EPC. 2011. The repertoire of ICE in prokaryotes underscores the unity, diversity, and ubiquity of conjugation. PLoS Genet 7:e1002222. doi:10.1371/journal.pgen.1002222 21876676 PMC3158045

[17] Delavat F, Miyazaki R, Carraro N, Pradervand N, van der Meer JR. 2017. The hidden life of integrative and conjugative elements. FEMS Microbiol Rev 41:512–537. doi:10.1093/femsre/fux008 28369623 PMC5812530

[18] Gonçalves OS, de Queiroz MV, Santana MF. 2020. Potential evolutionary impact of integrative and conjugative elements (ICEs) and genomic islands in the Ralstonia solanacearum species complex. Sci Rep 10:12498. doi:10.1038/s41598-020-69490-1 32719415 PMC7385641

[19] Gonçalves OS, Santana MF. 2021. The coexistence of monopartite integrative and conjugative elements in the genomes of Acidobacteria. Gene 777:145476. doi:10.1016/j.gene.2021.145476 33549716

[20] de Assis JCS, Gonçalves OS, Fernandes AS, de Queiroz MV, Bazzolli DMS, Santana MF. 2022. Genomic analysis reveals the role of integrative and conjugative elements in plant pathogenic bacteria. Mob DNA 13:19. doi:10.1186/s13100-022-00275-1 35962419 PMC9373382

[21] Burrus V, Waldor MK. 2004. Shaping bacterial genomes with integrative and conjugative elements. Res Microbiol 155:376–386. doi:10.1016/j.resmic.2004.01.012 15207870

[22] Johnson CM, Grossman AD. 2015. Integrative and conjugative elements (ICEs): what they do and how they work. Annu Rev Genet 49:577–601. doi:10.1146/annurev-genet-112414-055018 26473380 PMC5180612

[23] Nugent ME. 1981. A conjugative “plasmid” lacking autonomous replication. J Gen Microbiol 126:305–310. doi:10.1099/00221287-126-2-305 6279761

[24] Rashtchian A, Dubes GR, Booth SJ. 1982. Tetracycline-inducible transfer of tetracycline resistance in Bacteroides fragilis in the absence of detectable plasmid DNA. J Bacteriol 150:141–147. doi:10.1128/jb.150.1.141-147.1982 7061390 PMC220092

[25] Gaillard M, Vallaeys T, Vorhölter FJ, Minoia M, Werlen C, Sentchilo V, Pühler A, van der Meer JR. 2006. The clc element of Pseudomonas sp. strain B13, a genomic island with various catabolic properties. J Bacteriol 188:1999–2013. doi:10.1128/JB.188.5.1999-2013.2006 16484212 PMC1426575

[26] Obi CC, Vayla S, de Gannes V, Berres ME, Walker J, Pavelec D, Hyman J, Hickey WJ. 2018. The integrative conjugative element clc (ICEclc) of Pseudomonas aeruginosa JB2. Front Microbiol 9:1532. doi:10.3389/fmicb.2018.01532 30050515 PMC6050381

[27] Würdemann D, Tümmler B. 2007. In silico comparison of pKLC102-like genomic islands of Pseudomonas aeruginosa. FEMS Microbiol Lett 275:244–249. doi:10.1111/j.1574-6968.2007.00891.x 17714478

[28] Miyazaki R, Bertelli C, Benaglio P, Canton J, De Coi N, Gharib WH, Gjoksi B, Goesmann A, Greub G, Harshman K, Linke B, Mikulic J, Mueller L, Nicolas D, Robinson-Rechavi M, Rivolta C, Roggo C, Roy S, Sentchilo V, Siebenthal AV, Falquet L, van der Meer JR. 2015. Comparative genome analysis of Pseudomonas knackmussii B13, the first bacterium known to degrade chloroaromatic compounds. Environ Microbiol 17:91–104. doi:10.1111/1462-2920.12498 24803113

[29] Gaillard M, Pernet N, Vogne C, Hagenbüchle O, van der Meer JR. 2008. Host and invader impact of transfer of the clc genomic island into Pseudomonas aeruginosa PAO1. Proc Natl Acad Sci U S A 105:7058–7063. doi:10.1073/pnas.0801269105 18448680 PMC2383934

[30] Ravatn R, Zehnder AJ, van der Meer JR. 1998. Low-frequency horizontal transfer of an element containing the chlorocatechol degradation genes from Pseudomonas sp. strain B13 to Pseudomonas putida F1 and to indigenous bacteria in laboratory-scale activated-sludge microcosms. Appl Environ Microbiol 64:2126–2132. doi:10.1128/AEM.64.6.2126-2132.1998 9603824 PMC106288

[31] Springael D, Peys K, Ryngaert A, Van Roy S, Hooyberghs L, Ravatn R, Heyndrickx M, van der Meer JR, Vandecasteele C, Mergeay M, Diels L. 2002. Community shifts in a seeded 3-chlorobenzoate degrading membrane biofilm reactor: indications for involvement of in situ horizontal transfer of the clc-element from inoculum to contaminant bacteria. Environ Microbiol 4:70–80. doi:10.1046/j.1462-2920.2002.00267.x 11972616

[32] Pradervand N, Delavat F, Sulser S, Miyazaki R, van der Meer JR. 2014. The TetR-type MfsR protein of the integrative and conjugative element (ICE) ICEclc controls both a putative efflux system and initiation of ICE transfer. J Bacteriol 196:3971–3979. doi:10.1128/JB.02129-14 25182498 PMC4248833

[33] Carraro N, Richard X, Sulser S, Delavat F, Mazza C, van der Meer JR. 2020. An analog to digital converter controls bistable transfer competence development of a widespread bacterial integrative and conjugative element. Elife 9:e57915. doi:10.7554/eLife.57915 32720896 PMC7423338

[34] Minoia M, Gaillard M, Reinhard F, Stojanov M, Sentchilo V, van der Meer JR. 2008. Stochasticity and bistability in horizontal transfer control of a genomic island in Pseudomonas. Proc Natl Acad Sci U S A 105:20792–20797. doi:10.1073/pnas.0806164106 19098098 PMC2605633

[35] Ravatn R, Studer S, Zehnder AJ, van der Meer JR. 1998. Int-B13, an unusual site-specific recombinase of the bacteriophage P4 integrase family, is responsible for chromosomal insertion of the 105-kilobase clc element of Pseudomonas sp. strain B13. J Bacteriol 180:5505–5514. doi:10.1128/JB.180.21.5505-5514.1998 9791097 PMC107606

[36] Miyazaki R, van der Meer JR. 2011. A dual functional origin of transfer in the ICEclc genomic island of Pseudomonas knackmussii B13: oriT of ICEclc. Mol Microbiol 79:743–758. doi:10.1111/j.1365-2958.2010.07484.x 21255116

[37] Daveri A, Benigno V, van der Meer JR. 2023. Characterization of an atypical but widespread type IV secretion system for transfer of the integrative and conjugative element (ICEclc) in Pseudomonas putida. Nucleic Acids Res 51:2345–2362. doi:10.1093/nar/gkad024 36727472 PMC10018362

[38] Botelho J, Mourão J, Roberts AP, Peixe L. 2020. Comprehensive genome data analysis establishes a triple whammy of carbapenemases, ICEs and multiple clinically relevant bacteria. Microb Genom 6:10. doi:10.1099/mgen.0.000424 PMC7660259 32841111

[39] Botelho J, Grosso F, Peixe L. 2020. ICEs are the main reservoirs of the ciprofloxacin-modifying crpP gene in Pseudomonas aeruginosa. Genes 11:889. doi:10.3390/genes11080889 32759827 PMC7463715

[40] Botelho J, Schulenburg H. 2021. The role of integrative and conjugative elements in antibiotic resistance evolution. Trends Microbiol 29:8–18. doi:10.1016/j.tim.2020.05.011 32536522

[41] Nikolaou E, Hubbard ATM, Botelho J, Marschall TAM, Ferreira DM, Roberts AP. 2020. Antibiotic resistance is associated with integrative and conjugative elements and genomic islands in naturally circulating Streptococcus pneumoniae isolates from adults in Liverpool, UK. Genes (Basel) 11:625. doi:10.3390/genes11060625 32517221 PMC7348760

[42] Hong JS, Yoon E-J, Lee H, Jeong SH, Lee K. 2016. Clonal dissemination of Pseudomonas aeruginosa sequence type 235 isolates carrying bla_IMP-6_ and emergence of bla_GES-24_ and bla_IMP-10_ on novel genomic islands PAGI-15 and -16 in South Korea. Antimicrob Agents Chemother 60:7216–7223. doi:10.1128/AAC.01601-16 27671068 PMC5119001

[43] Brizuela J, Kajeekul R, Roodsant TJ, Riwload A, Boueroy P, Pattanapongpaibool A, Thaipadungpanit J, Jenjaroenpun P, Wongsurawat T, Batty EM, van der Putten BCL, Schultsz C, Kerdsin A. 2023. Streptococcus suis outbreak caused by an emerging zoonotic strain with acquired multi-drug resistance in Thailand. Microb Genom 9:mgen000952. doi:10.1099/mgen.0.000952 36790403 PMC9997742

[44] Botelho J, Tüffers L, Fuss J, Buchholz F, Utpatel C, Klockgether J, Niemann S, Tümmler B, Schulenburg H. 2023. Phylogroup-specific variation shapes the clustering of antimicrobial resistance genes and defence systems across regions of genome plasticity in Pseudomonas aeruginosa. EBioMedicine 90:104532. doi:10.1016/j.ebiom.2023.104532 36958270 PMC10053402

[45] Slekovec C, Robert J, van der Mee-Marquet N, Berthelot P, Rogues A-M, Derouin V, Cholley P, Thouverez M, Hocquet D, Bertrand X. 2019. Molecular epidemiology of Pseudomonas aeruginosa isolated from infected ICU patients: a French multicenter 2012–2013 study. Eur J Clin Microbiol Infect Dis 38:921–926. doi:10.1007/s10096-019-03519-w 30826996

[46] Oliver A, Mulet X, López-Causapé C, Juan C. 2015. The increasing threat of Pseudomonas aeruginosa high-risk clones. Drug Resist Updat 21–22:41–59. doi:10.1016/j.drup.2015.08.002 26304792

[47] Pradervand N, Sulser S, Delavat F, Miyazaki R, Lamas I, van der Meer JR. 2014. An Operon of three transcriptional regulators controls horizontal gene transfer of the integrative and Conjugative element ICEclc in Pseudomonas knackmussii B13. PLoS genetics 10:e1004441. doi:10.1371/journal.pgen.1004441 24945944 PMC4063739

[48] Qiu X, Gurkar AU, Lory S. 2006. Interstrain transfer of the large pathogenicity island (PAPI-1) of Pseudomonas aeruginosa. Proc Natl Acad Sci U S A 103:19830–19835. doi:10.1073/pnas.0606810104 17179047 PMC1750864

[49] Takano S, Fukuda K, Koto A, Miyazaki R. 2019. A novel system of bacterial cell division arrest implicated in horizontal transmission of an integrative and conjugative element. PLoS Genet 15:e1008445. doi:10.1371/journal.pgen.1008445 31609967 PMC6812849

[50] Francia MV, Varsaki A, Garcillán-Barcia MP, Latorre A, Drainas C, de la Cruz F. 2004. A classification scheme for mobilization regions of bacterial plasmids. FEMS Microbiol Rev 28:79–100. doi:10.1016/j.femsre.2003.09.001 14975531

[51] Carraro N, Rivard N, Burrus V, Ceccarelli D. 2017. Mobilizable genomic islands, different strategies for the dissemination of multidrug resistance and other adaptive traits. Mob Genet Elements 7:1–6. doi:10.1080/2159256X.2017.1304193 PMC5397120 28439449

[52] Tettelin H, Masignani V, Cieslewicz MJ, Donati C, Medini D, Ward NL, Angiuoli SV, Crabtree J, Jones AL, Durkin AS, et al.. 2005. Genome analysis of multiple pathogenic isolates of Streptococcus agalactiae: implications for the microbial “pan-genome". Proc Natl Acad Sci U S A 102:13950–13955. doi:10.1073/pnas.0506758102 16172379 PMC1216834

[53] Millman A, Melamed S, Amitai G, Sorek R. 2020. Diversity and classification of cyclic-oligonucleotide-based anti-phage signalling systems. Nat Microbiol 5:1608–1615. doi:10.1038/s41564-020-0777-y 32839535 PMC7610970

[54] Ye Q, Lau RK, Mathews IT, Birkholz EA, Watrous JD, Azimi CS, Pogliano J, Jain M, Corbett KD. 2020. HORMA domain proteins and a Trip13-like ATPase regulate bacterial cGAS-like enzymes to mediate bacteriophage immunity. Mol Cell 77:709–722. doi:10.1016/j.molcel.2019.12.009 31932165 PMC7036143

[55] Masuda N, Sakagawa E, Ohya S, Gotoh N, Tsujimoto H, Nishino T. 2000. Contribution of the MexX-MexY-OprM efflux system to intrinsic resistance in Pseudomonas aeruginosa. Antimicrob Agents Chemother 44:2242–2246. doi:10.1128/AAC.44.9.2242-2246.2000 10952562 PMC90052

[56] Morita Y, Tomida J, Kawamura Y. 2012. MexXY multidrug efflux system of Pseudomonas aeruginosa. Front Microbiol 3:408. doi:10.3389/fmicb.2012.00408 23233851 PMC3516279

[57] Dantas RCC, Silva RTE, Ferreira ML, Gonçalves IR, Araújo BF, Campos P de, Royer S, Batistão D da F, Gontijo-Filho PP, Ribas RM. 2017. Molecular epidemiological survey of bacteremia by multidrug resistant Pseudomonas aeruginosa: the relevance of intrinsic resistance mechanisms. PLoS One 12:e0176774. doi:10.1371/journal.pone.0176774 28481953 PMC5421754

[58] Li X-Z, Elkins CA, Zgurskaya HI. 2016. Efflux-mediated antimicrobial resistance in bacteria, p 359–400. In Antimicrobial drug efflux pumps in Pseudomonas aeruginosa. Springer International Publishing, Cham. doi:10.1007/978-3-319-39658-3

[59] Zahedi Bialvaei A, Rahbar M, Hamidi-Farahani R, Asgari A, Esmailkhani A, Mardani Dashti Y, Soleiman-Meigooni S. 2021. Expression of RND efflux pumps mediated antibiotic resistance in Pseudomonas aeruginosa clinical strains. Microb Pathog 153:104789. doi:10.1016/j.micpath.2021.104789 33556480

[60] Kamal SM, Rybtke ML, Nimtz M, Sperlein S, Giske C, Trček J, Deschamps J, Briandet R, Dini L, Jänsch L, Tolker-Nielsen T, Lee C, Römling U. 2019. Two FtsH proteases contribute to fitness and adaptation of Pseudomonas aeruginosa clone C strains. Front Microbiol 10:1372. doi:10.3389/fmicb.2019.01372 31338071 PMC6629908

[61] Basta DW, Angeles-Albores D, Spero MA, Ciemniecki JA, Newman DK. 2020. Heat-shock proteases promote survival of Pseudomonas aeruginosa during growth arrest. Proc Natl Acad Sci U S A 117:4358–4367. doi:10.1073/pnas.1912082117 32029587 PMC7049150

[62] Sulser S, Vucicevic A, Bellini V, Moritz R, Delavat F, Sentchilo V, Carraro N, van der Meer JR. 2022. A bistable prokaryotic differentiation system underlying development of conjugative transfer competence. PLoS Genet 18:e1010286. doi:10.1371/journal.pgen.1010286 35763548 PMC9286271

[63] Romano S, Bourdier A, Parer S, Masnou A, Burgel L, Raczka F, Lamy B, Jumas-Bilak E, Lotthé A. 2013. Peripheral venous catheter and bloodstream infection caused by Pseudomonas aeruginosa after a contaminated preoperative shower. Infect Control Hosp Epidemiol 34:544–546. doi:10.1086/670221 23571380

[64] Virieux-Petit M, Hammer-Dedet F, Aujoulat F, Jumas-Bilak E, Romano-Bertrand S. 2022. From copper tolerance to resistance in Pseudomonas aeruginosa towards patho-adaptation and hospital success. Genes (Basel) 13:301. doi:10.3390/genes13020301 35205346 PMC8872213

[65] Bédard E, Prévost M, Déziel E. 2016. Pseudomonas aeruginosa in premise plumbing of large buildings. Microbiologyopen 5:937–956. doi:10.1002/mbo3.391 27353357 PMC5221438

[66] Loveday HP, Wilson JA, Kerr K, Pitchers R, Walker JT, Browne J. 2014. Association between healthcare water systems and Pseudomonas aeruginosa infections: a rapid systematic review. J Hosp Infect 86:7–15. doi:10.1016/j.jhin.2013.09.010 24289866

[67] Blanc DS, Gomes Magalhaes B, Abdelbary M, Prod’hom G, Greub G, Wasserfallen JB, Genoud P, Zanetti G, Senn L. 2016. Hand soap contamination by Pseudomonas aeruginosa in a tertiary care hospital: no evidence of impact on patients. J Hosp Infect 93:63–67. doi:10.1016/j.jhin.2016.02.010 27021398

[68] Magalhães B, Valot B, Abdelbary MMH, Prod’hom G, Greub G, Senn L, Blanc DS. 2020. Combining standard molecular typing and whole genome sequencing to investigate Pseudomonas aeruginosa epidemiology in intensive care units. Front Public Health 8:3. doi:10.3389/fpubh.2020.00003 32047733 PMC6997133

[69] Tissot F, Blanc DS, Basset P, Zanetti G, Berger MM, Que Y-A, Eggimann P, Senn L. 2016. New genotyping method discovers sustained nosocomial Pseudomonas aeruginosa outbreak in an intensive care burn unit. J Hosp Infect 94:2–7. doi:10.1016/j.jhin.2016.05.011 27451039

[70] Cholley P, Stojanov M, Hocquet D, Thouverez M, Bertrand X, Blanc DS. 2015. Comparison of double-locus sequence typing (DLST) and multilocus sequence typing (MLST) for the investigation of Pseudomonas aeruginosa populations. Diagn Microbiol Infect Dis 82:274–277. doi:10.1016/j.diagmicrobio.2015.03.027 25952482

[71] Catho G, Martischang R, Boroli F, Chraïti MN, Martin Y, Koyluk Tomsuk Z, Renzi G, Schrenzel J, Pugin J, Nordmann P, Blanc DS, Harbarth S. 2021. Outbreak of Pseudomonas aeruginosa producing VIM carbapenemase in an intensive care unit and its termination by implementation of waterless patient care. Crit Care 25:301. doi:10.1186/s13054-021-03726-y 34412676 PMC8376114

[72] Andrews S. 2010. A quality control tool for high throughput sequence data. Available from: https://www.bioinformatics.babraham.ac.uk/projects/fastqc/

[73] Bolger AM, Lohse M, Usadel B. 2014. Trimmomatic: a flexible trimmer for Illumina sequence data. Bioinformatics 30:2114–2120. doi:10.1093/bioinformatics/btu170 24695404 PMC4103590

[74] Nurk S, Bankevich A, Antipov D, Gurevich A, Korobeynikov A, Lapidus A, Prjibelsky A, Pyshkin A, Sirotkin A, Sirotkin Y, Stepanauskas R, McLean J, Lasken R, Clingenpeel SR, Woyke T, Tesler G, Alekseyev MA, Pevzner PA. 2013. Assembling genomes and mini-metagenomes from highly chimeric reads, p 158–170. In Deng M, R Jiang, F Sun, X Zhang (ed), Research in computational molecular biology. doi:10.1007/978-3-642-37195-0_13 PMC3791033 24093227

[75] Alonge M, Lebeigle L, Kirsche M, Aganezov S, Wang X, Lippman ZB, Schatz MC, Soyk S. 2021. Automated assembly scaffolding elevates a new tomato system for high-throughput genome editing. Plant Biology. doi:10.1101/2021.11.18.469135 PMC9753292 36522651

[76] Seemann T. n.d. MLST: scan contig files against traditional PubMLST typing schemes. Available from: https://github.com/tseemann/mlst

[77] Jolley KA, Maiden MCJ. 2010. BIGSdb: scalable analysis of bacterial genome variation at the population level. BMC Bioinformatics 11:595. doi:10.1186/1471-2105-11-595 21143983 PMC3004885

[78] Zhang Z, Schwartz S, Wagner L, Miller W. 2000. A greedy algorithm for aligning DNA sequences. J Comput Biol 7:203–214. doi:10.1089/10665270050081478 10890397

[79] Shen W, Le S, Li Y, Hu F. 2016. SeqKit: a cross-platform and ultrafast toolkit for FASTA/Q file manipulation. PLoS One 11:e0163962. doi:10.1371/journal.pone.0163962 27706213 PMC5051824

[80] Sentchilo V, Czechowska K, Pradervand N, Minoia M, Miyazaki R, van der Meer JR. 2009. Intracellular excision and reintegration dynamics of the ICEclc genomic island of Pseudomonas knackmussii sp. strain B13. Mol Microbiol 72:1293–1306. doi:10.1111/j.1365-2958.2009.06726.x 19432799

[81] Seemann T. 2014. Prokka: rapid prokaryotic genome annotation. Bioinformatics 30:2068–2069. doi:10.1093/bioinformatics/btu153 24642063

[82] Guy L, Kultima JR, Andersson SGE. 2010. genoPlotR: comparative gene and genome visualization in R. Bioinformatics 26:2334–2335. doi:10.1093/bioinformatics/btq413 20624783 PMC2935412

[83] Chan PP, Lowe TM. 2019. tRNAscan-SE: searching for tRNA genes in genomic sequences, p 1–14. In Kollmar M (ed), Gene prediction. Springer New York. doi:10.1007/978-1-4939-9173-0 PMC6768409 31020551

[84] Garcillán-Barcia MP, Redondo-Salvo S, Vielva L, Cruz F. 2020. MOBscan: automated annotation of MOB relaxases, p 295–308. In de la Cruz F (ed), Horizontal gene transfer. Springer US, New York. doi:10.1007/978-1-4939-9877-7 31584171

[85] Jain C, Rodriguez-R LM, Phillippy AM, Konstantinidis KT, Aluru S. 2018. High throughput ANI analysis of 90K prokaryotic genomes reveals clear species boundaries. Nat Commun 9:5114. doi:10.1038/s41467-018-07641-9 30504855 PMC6269478

[86] Grant BJ, Rodrigues APC, ElSawy KM, McCammon JA, Caves LSD. 2006. Bio3d: an R package for the comparative analysis of protein structures. Bioinformatics 22:2695–2696. doi:10.1093/bioinformatics/btl461 16940322

[87] Liu M, Li X, Xie Y, Bi D, Sun J, Li J, Tai C, Deng Z, Ou H-Y. 2019. ICEberg 2.0: an updated database of bacterial integrative and conjugative elements. Nucleic Acids Res 47:D660–D665. doi:10.1093/nar/gky1123 30407568 PMC6323972

[88] Contreras-Moreira B, Vinuesa P. 2013. GET_HOMOLOGUES, a versatile software package for scalable and robust microbial pangenome analysis. Appl Environ Microbiol 79:7696–7701. doi:10.1128/AEM.02411-13 24096415 PMC3837814

[89] Li L, Stoeckert CJ, Roos DS. 2003. OrthoMCL: identification of ortholog groups for eukaryotic genomes. Genome Res 13:2178–2189. doi:10.1101/gr.1224503 12952885 PMC403725

[90] Emms DM, Kelly S. 2019. OrthoFinder: phylogenetic orthology inference for comparative genomics. Genome Biol 20:238. doi:10.1186/s13059-019-1832-y 31727128 PMC6857279

[91] Katoh K. 2002. MAFFT: a novel method for rapid multiple sequence alignment based on fast fourier transform. Nucleic Acids Res 30:3059–3066. doi:10.1093/nar/gkf436 12136088 PMC135756

[92] Minh BQ, Schmidt HA, Chernomor O, Schrempf D, Woodhams MD, von Haeseler A, Lanfear R. 2020. IQ-TREE 2: new models and efficient methods for phylogenetic inference in the genomic era. Mol Biol Evol 37:1530–1534. doi:10.1093/molbev/msaa131 32011700 PMC7182206

[93] Simon T, Robert MM. 1986. Lectures on mathematics in the life sciences, p 57–86. American Mathematical Society.

[94] Hasegawa M, Kishino H, Yano T. 1985. Dating of the human-ape splitting by a molecular clock of mitochondrial DNA. J Mol Evol 22:160–174. doi:10.1007/BF02101694 3934395

[95] Yu G, Smith DK, Zhu H, Guan Y, Lam TT, McInerny G. 2017. GGTREE: an R package for visualization and annotation of phylogenetic trees with their covariates and other associated data. Methods Ecol Evol 8:28–36. doi:10.1111/2041-210X.12628

[96] Galili T. 2015. dendextend: an R package for visualizing, adjusting and comparing trees of hierarchical clustering. Bioinformatics 31:3718–3720. doi:10.1093/bioinformatics/btv428 26209431 PMC4817050

[97] Altenhoff AM, Levy J, Zarowiecki M, Tomiczek B, Warwick Vesztrocy A, Dalquen DA, Müller S, Telford MJ, Glover NM, Dylus D, Dessimoz C. 2019. OMA standalone: orthology inference among public and custom genomes and transcriptomes. Genome Res 29:1152–1163. doi:10.1101/gr.243212.118 31235654 PMC6633268

[98] Hadfield J, Croucher NJ, Goater RJ, Abudahab K, Aanensen DM, Harris SR. 2018. Phandango: an interactive viewer for bacterial population genomics. Bioinformatics 34:292–293. doi:10.1093/bioinformatics/btx610 29028899 PMC5860215

[99] Wu S, Zhu Z, Fu L, Niu B, Li W. 2011. WebMGA: a customizable web server for fast metagenomic sequence analysis. BMC Genomics 12:444. doi:10.1186/1471-2164-12-444 21899761 PMC3180703

