# Supplementary figures and images for: Diversity and evolution of an abundant ICEclc family of integrative and conjugative elements in Pseudomonas aeruginosa
Source: mSphere. 2023 Oct 30;8(6):e00517-23. doi: 10.1128/msphere.00517-23 (PMC10732049; doi:10.1128/msphere.00517-23)

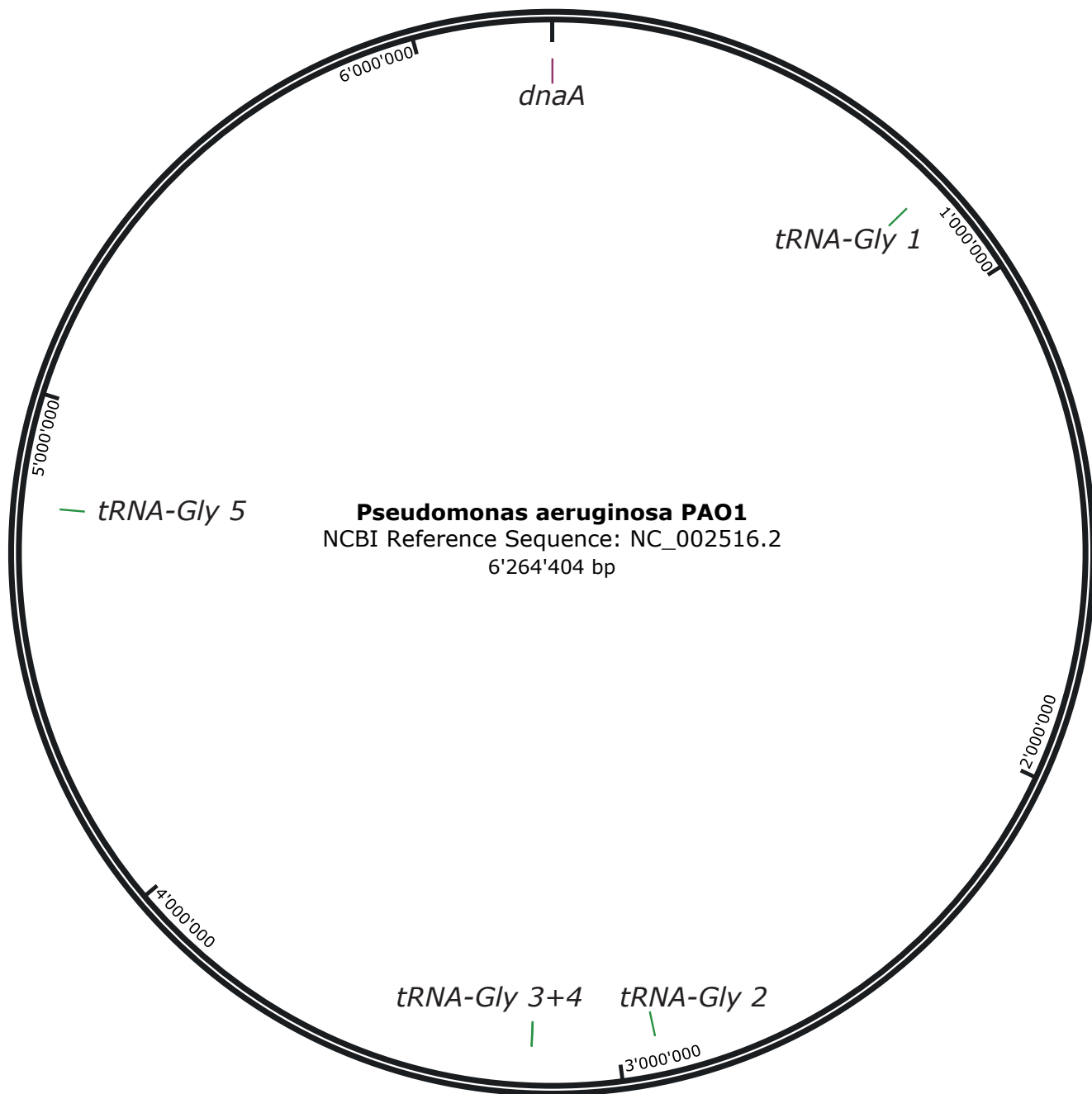

**Pseudomonas aeruginosa PAO1**  
NCBI Reference Sequence: NC\_002516.2  
6'264'404 bp

Supplement: Fig. S1 — Chromosome map of P. aeruginosa PAO1. [file msphere.00517-23-s0001.pdf]

ICE clone ID

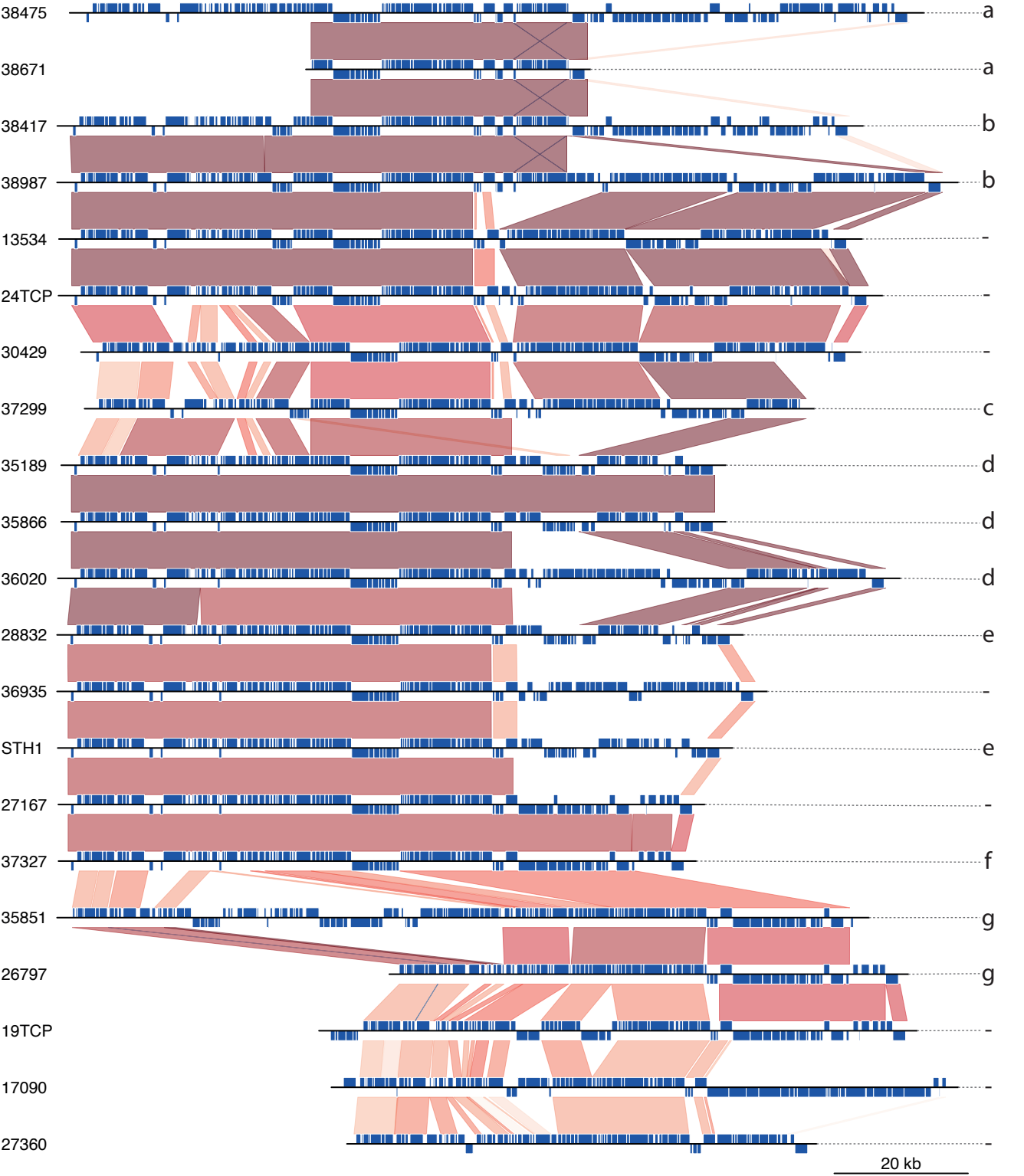

Supplement: Fig. S2 — Gene synteny comparisons among the 21 P. aeruginosa ICEclc-family elements. [file msphere.00517-23-s0002.pdf]

## Integrase gene tree

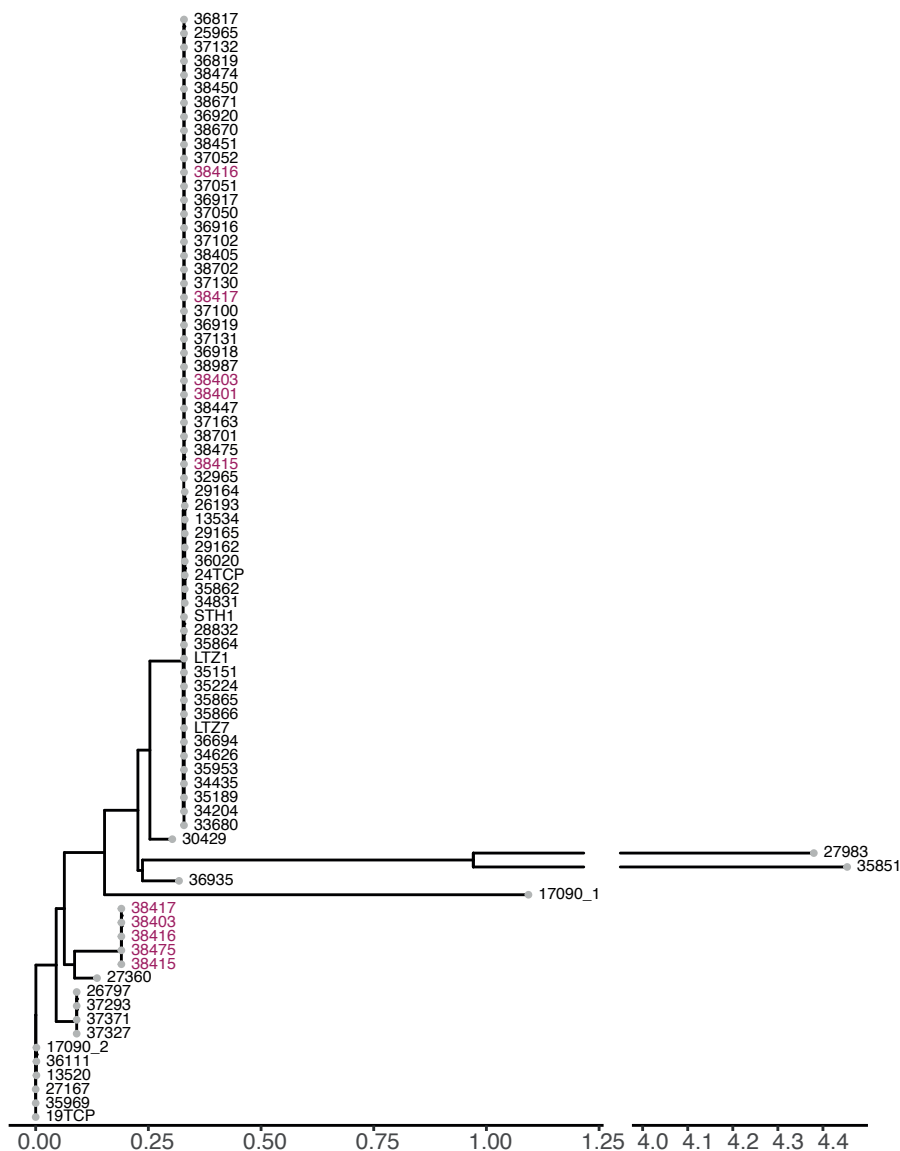

### Percentage nucleotide identity (integrase gene)

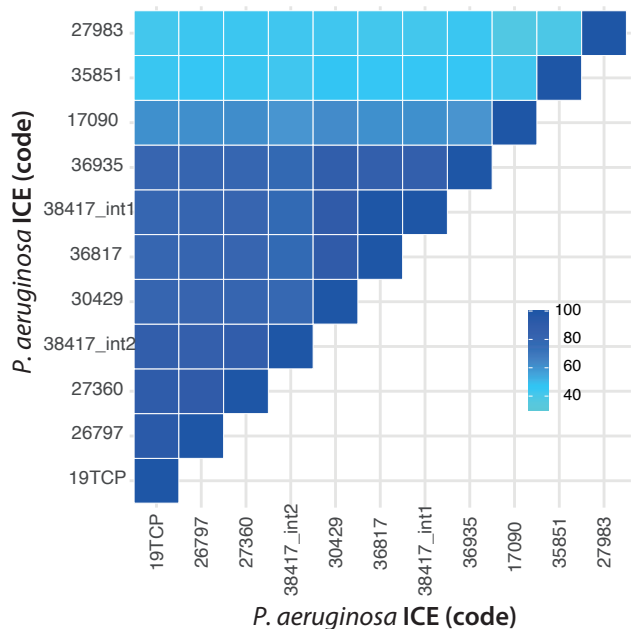

Supplement: Fig. S3 — ICE relatedness inferred from similarities among their integrases. [file msphere.00517-23-s0003.pdf]
