# Supplementary material for: Diversity and evolution of an abundant ICEclc family of integrative and conjugative elements in Pseudomonas aeruginosa
Source: mSphere. 2023 Oct 30;8(6):e00517-23. doi: 10.1128/msphere.00517-23 (PMC10732049; doi:10.1128/msphere.00517-23)
Supplement: Captions — to all supplemental material. [file msphere.00517-23-s0004.docx]

**Supplementary Fig. 1:** **Chromosome map of *P. aeruginosa* PAO1.** Circulat map (NCBI accession number NC_002516.2) showing the order of the five genes coding for tRNA-Gly relative to the *dnaA* gene. Sixty-eight ICEs identified in our study have *att* sequences corresponding to the 3’-end 18-bp of the second, third and fourth copy of the *tRNA-Gly* gene in the *dnaA*-aligned reference genome (as listed in Table S2). One element (ICE_17090) contains a different set of direct repeats and is integrated into the *tRNA-Gly*–1 gene.

**Supplementary Fig. 2: Gene synteny comparisons among the 21 *P. aeruginosa* ICE*clc*-family elements.** ICE elements selected from Figure 2A, in red, with their sub-group assignment indicated with letters a-g). ORFs and their orientation are represented by blue boxes on top (forward) or bottom (reverse strand). Blocks in between ICEs indicate regions of significant BLASTN homology (hits above default BLASTN thresholds, according to color-scale, with darker colors indicating higher homology). General conservation of a ‘core’ region is clearly visible, as well as individual micro deletions and insertions.

**Supplementary Fig. 3: ICE relatedness inferred from similarities among their integrases**. A) Consensus (n=1000 bootstraps) integrase gene tree based on nucleotide alignment. ICEs that contain two (different) integrases are highlighted with magenta IDs. B) Paired nucleotide similarities (as percentage identity, according to color scale) of 11 representative integrases (one from each sub-tree in panel A) from a multiple sequence alignment over the complete sequence.

**Supplementary Table 1: Characteristics of *Pseudomonas aeruginosa* isolates.**

**Supplementary Table 2: Characteristics of the attributed ICE*clc* family elements in individual *Pseudomonas aeruginosa* isolates from the regional hospital.**

**Supplementary Table 3: Total attribution of integrative and conjugative/mobilizable elements in *P. aeruginosa* isolates from the regional hospital.**

**Supplementary Table 4: Deduced core functions of ICE*clc* family members**

**Supplementary Table 5: Gene function occurrences among the ICE*clc* family gene 'cloud' in regional *P. aeruginosa* clinical isolates**

**Supplementary Table 6: ICE*clc* family members identified among a world-wide survey of publicly available *P. aeruginosa* genomes.**
